# PTEN deficiency promotes pathological vascular remodeling of human coronary arteries

**DOI:** 10.1172/jci.insight.97228

**Published:** 2018-02-22

**Authors:** Karen S. Moulton, Marcella Li, Keith Strand, Shawna Burgett, Penn McClatchey, Rebecca Tucker, Seth B. Furgeson, Sizhao Lu, Bruce Kirkpatrick, Joseph C. Cleveland, Raphael A. Nemenoff, Amrut V. Ambardekar, Mary C.M. Weiser-Evans

**Affiliations:** 1 Division of Cardiology, Department of Medicine,; 2 Division of Renal Diseases and Hypertension, Department of Medicine,; 3 Graduate School Bioengineering Graduate Program,; 4 School of Medicine, Consortium for Fibrosis Research and Translation,; 5 Department of Surgery,; 6 Cardiovascular Pulmonary Research Program, University of Colorado Anschutz Medical Campus, Aurora, Colorado, USA.

**Keywords:** Vascular Biology, Cardiovascular disease

## Abstract

Phosphatase and tensin homolog (PTEN) is an essential regulator of the differentiated vascular smooth muscle cell (SMC) phenotype. Our goal was to establish that PTEN loss promotes SMC dedifferentiation and pathological vascular remodeling in human atherosclerotic coronary arteries and nonatherosclerotic coronary arteries exposed to continuous-flow left ventricular assist devices (CF-LVADs). Arteries were categorized as nonatherosclerotic hyperplasia (NAH), atherosclerotic hyperplasia (AH), or complex plaque (CP). NAH coronary arteries from CF-LVAD patients were compared to NAH coronaries from non-LVAD patients. Intimal PTEN and SMC contractile protein expression was reduced compared with the media in arteries with NAH, AH, or CP. Compared with NAH, PTEN and SMC contractile protein expression was reduced in the media and intima of arteries with AH and CP. NAH arteries from CF-LVAD patients showed marked vascular remodeling and reduced PTEN and α-smooth muscle actin (αSMA) in medial SMCs compared with arteries from non-LVAD patients; this correlated with increased medial collagen deposition. Mechanistically, compared with ApoE^–/–^ mice, SMC-specific PTEN-null/ApoE^–/–^ double-knockout mice exhibited accelerated atherosclerosis progression and increased vascular fibrosis. By microarray and validated quantitative RT-PCR analysis, SMC PTEN deficiency promotes a global upregulation of proinflammatory and profibrotic genes. We propose that PTEN is an antiinflammatory, antifibrotic target that functions to maintain SMC differentiation. SMC loss of PTEN results in pathological vascular remodeling of human arteries.

## Abstract 2

PTEN loss correlates with dedifferentiation of smooth muscle cells of human coronary arteries affected with atherosclerosis or exposed to continuous-flow left ventricular assist devices.

## Introduction

Atherosclerosis, well recognized as a chronic inflammatory disease that progresses to complex, unstable arterial lesions, is the underlying cause of cardiovascular disease–related deaths due to myocardial infarction, cerebral vascular accident, peripheral artery disease, and ischemic cardiomyopathy (1–4). Atherosclerosis is a progressive, multifactorial disease in which multiple cell types, including vascular smooth muscle cells (SMCs) and immune cells of both the innate and adaptive immune systems, contribute to the pathology (2, 5). One major characteristic feature of atherosclerosis is dedifferentiation of vascular SMCs, contributing to intimal lesion formation and vessel occlusion (5–8). Differentiated SMCs are highly specialized cells whose primary role is to maintain vessel homeostasis, vessel tone, blood pressure, and blood flow distribution (5). This function is driven through expression of SMC-specific contractile and contractile-related proteins, including smooth muscle myosin heavy chain (SMMHC/*Myh11*), α-smooth muscle actin (αSMA/*Acta2*), SM22α (*Tagln1*), and calponin (*Cnn1*), among others. Unlike terminally differentiated cardiac and skeletal muscle, SMCs retain a significant degree of phenotypic plasticity and exhibit the ability to undergo extensive changes in phenotype in response to specific stimuli (i.e., dedifferentiated SMCs) (5–8). SMC dedifferentiation is associated with a transition to a highly proliferative, inflammatory phenotype characterized by downregulation of SMC-specific genes and increased production of multiple inflammatory and extracellular matrix–associated (ECM-associated) mediators (5–9). Thus, SMCs are major contributors to pathological vascular remodeling and vascular disease progression, and defining molecular mechanisms regulating SMC phenotypic transitions is critical to identify novel therapeutics for the treatment or regression of atherosclerosis.

The use of continuous-flow left ventricular assist devices (CF-LVADs) has increasingly replaced older-generation pulsatile-flow LVADs as a bridge to transplant or as a destination therapy for end-stage heart failure patients (10). While significant advances in survival have been achieved with the use of CF-LVADs, there continues to be an ongoing risk of adverse events, including ventricular arrhythmias, gastrointestinal bleeding, thromboembolic events, renal impairment, and hypertension (11–13). Emerging data suggest that alterations in vascular function and structure are associated with the nonphysiological systemic blood flow that results from the continuous, nonpulsatile flow generated by CF-LVADs (13–17). These vascular structural changes likely contribute to some of the complications after CF-LVAD placement. Several studies from our group and others have reported altered aortic morphology in CF-LVAD patients, including increased collagen deposition, elastin fragmentation, and SMC phenotypic changes, which contribute to decreased vessel compliance and increased vessel stiffness. In addition, peripheral vascular dysfunction and multiorgan failure have been observed in CF-LVAD patients (18, 19). The underlying molecular mechanisms associated with these functional and morphological changes and the role of SMCs in CF-LVAD–associated vascular remodeling, however, are largely unknown.

Regulation of the SMC differentiated phenotype is complex, involving multiple signaling pathways and transcriptional regulators (5, 8, 20). Most SMC-specific genes are under transcriptional control by the transcription factor, serum response factor (SRF), and its cardiac and SMC-specific cofactor, myocardin (21–27). We recently demonstrated that the tumor suppressor, phosphatase and tensin homolog (PTEN), interacts with SRF and myocardin in the nucleus and functions as an indispensable cofactor to maintain the differentiated SMC phenotype, a novel and phosphatase-independent function for PTEN (28). PTEN classically functions as a dual-specificity phosphatase to suppress numerous signaling networks involved in cell proliferation, inflammation, and ECM remodeling (29, 30). Our previous work in cell culture and mouse models of vascular disease demonstrated that loss of PTEN, and in particular nuclear PTEN, promotes reprogramming of SMCs into a dedifferentiated, proliferative, inflammatory phenotype (28, 31–33). Further, in a small cohort of human atherosclerotic coronary arteries, we reported decreased expression of PTEN in intimal SMCs (28). However, whether progressive loss of PTEN in SMCs is associated with severity of atherosclerosis in human coronary arteries is unknown. In addition, whether decreased SMC PTEN expression correlates with vascular remodeling and fibrosis observed in nonatherosclerotic arteries from CF-LVAD patients has not been studied. Therefore, the purpose of this translational study was to determine if changes in PTEN expression correlated to SMC dedifferentiation and pathological vascular remodeling in human atherosclerotic coronary arteries as well as nonatherosclerotic coronary arteries exposed to CF-LVADs.

## Results

The clinical variables for human subjects and coronary arteries used in this study are summarized in Table 1. The age, gender mix, blood pressure, and heart rate of individuals in the nonatherosclerotic hyperplasia (NAH; age range 24–65; 38.5% female), atherosclerotic hyperplasia (AH; age range 24–69; 40% female), complex plaque (CP; age range 43–63; 20% female), and nonatherosclerotic LVAD (NAH+LVAD; age range 43–63; 29.4% female) groups were similar. The mean duration of CF-LVAD was 206 days (range 26–653). The only difference in medications was an increased incidence of angiotensin-converting enzyme inhibitor or angiotensin receptor blocker in NAH+LVAD patients prior to heart transplant. The description of each vessel sample, the age, sex, and heart failure etiology of the patient source, and the individual assays performed on each vessel are listed in Supplemental Tables 2–5 (supplemental material available online with this article; https://doi.org/10.1172/jci.insight.97228DS1).

### Decreased PTEN and αSMA expression in human atherosclerotic coronary arteries.

**Figure 1:**
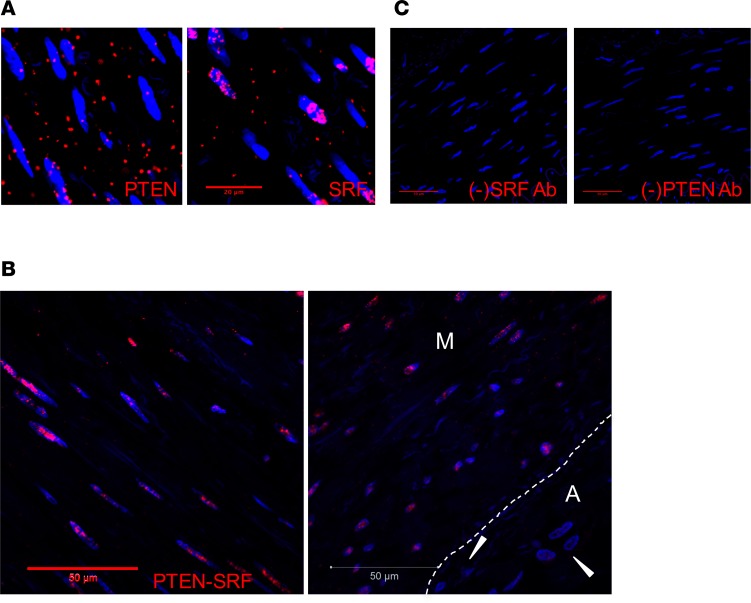
Interaction of PTEN and serum response factor (SRF) in the nucleus of medial smooth muscle cells (SMCs). Proximity ligation assay (PLA) and confocal microscopy were used to detect PTEN-SRF interactions in medial SMCs of human nonatherosclerotic coronary arteries. Representative images shown. (**A**) Left: PTEN positive control using mouse anti-PTEN primary antibody and anti-mouse PLUS and anti-mouse MINUS PLA probe demonstrates nuclear and cytoplasmic expression of PTEN. Right: SRF positive control using rabbit anti-SRF primary antibody and anti-rabbit PLUS and anti-rabbit MINUS PLA probe demonstrates predominantly nuclear expression of SRF. Scale bar: 20 μm. (**B**) PLA using mouse anti-PTEN and rabbit anti-SRF primary antibodies and anti-mouse PLUS and anti-rabbit MINUS PLA probe demonstrates PTEN-SRF nuclear interactions in medial SMCs of human nonatherosclerotic hyperplasia coronary arteries, but no interactions in adventitial cells (arrowheads; right panel). M = media; A = adventitia; white dashed line in right panel indicates the external elastic lamina. Scale bars: 50 μm. (**C**) PLA negative controls for SRF (left) and PTEN (right) demonstrate lack of signal when either primary antibodies are omitted. Scale bars: 50 μm. For all panels: Red = positive PLA; Blue = DAPI for cell nuclei.

Using cell culture and genetic mouse models, our previous work demonstrated that PTEN interacts with the transcription factor SRF in the nucleus of differentiated SMCs to facilitate SRF-dependent SMC gene transcription and regulation of the differentiated SMC phenotype (28). We used a proximity ligation assay (PLA) approach to determine if this interaction is also observed in medial SMCs of nonatherosclerotic human coronary arteries. As expected, PTEN was detected in both the cytoplasm and nucleus, whereas SRF was predominantly detected in the nucleus when using species-specific PLUS and MINUS PLA probes and single antigen-specific antibodies (Figure 1A). Consistent with our previous data, using both primary antibodies and species-specific PLUS and MINUS PLA probes, the proximity interaction between PTEN and SRF was detected predominantly in the nucleus of medial SMCs (Figure 1B and Supplemental Figure 1, A–G). No positive interaction (PLA signal) was detected in adventitial cells (Figure 1B), except for SMCs of adventitial vasa vasora (Supplemental Figure 1H). The percentage of medial SMCs expressing positive nuclear PLA signals was quantified in a small cohort of nonatherosclerotic vessels (Supplemental Figure 2I; *N* = 4 vessels from *N* = 4 patients). No interaction was detected when either primary antibody was omitted from the reaction (Figure 1C). Therefore, these data in human tissues strengthen our previous findings that PTEN interacts with SRF in SMCs and functions as an essential regulator of SRF-dependent transcriptional control of SMC differentiation.

**Figure 2:**
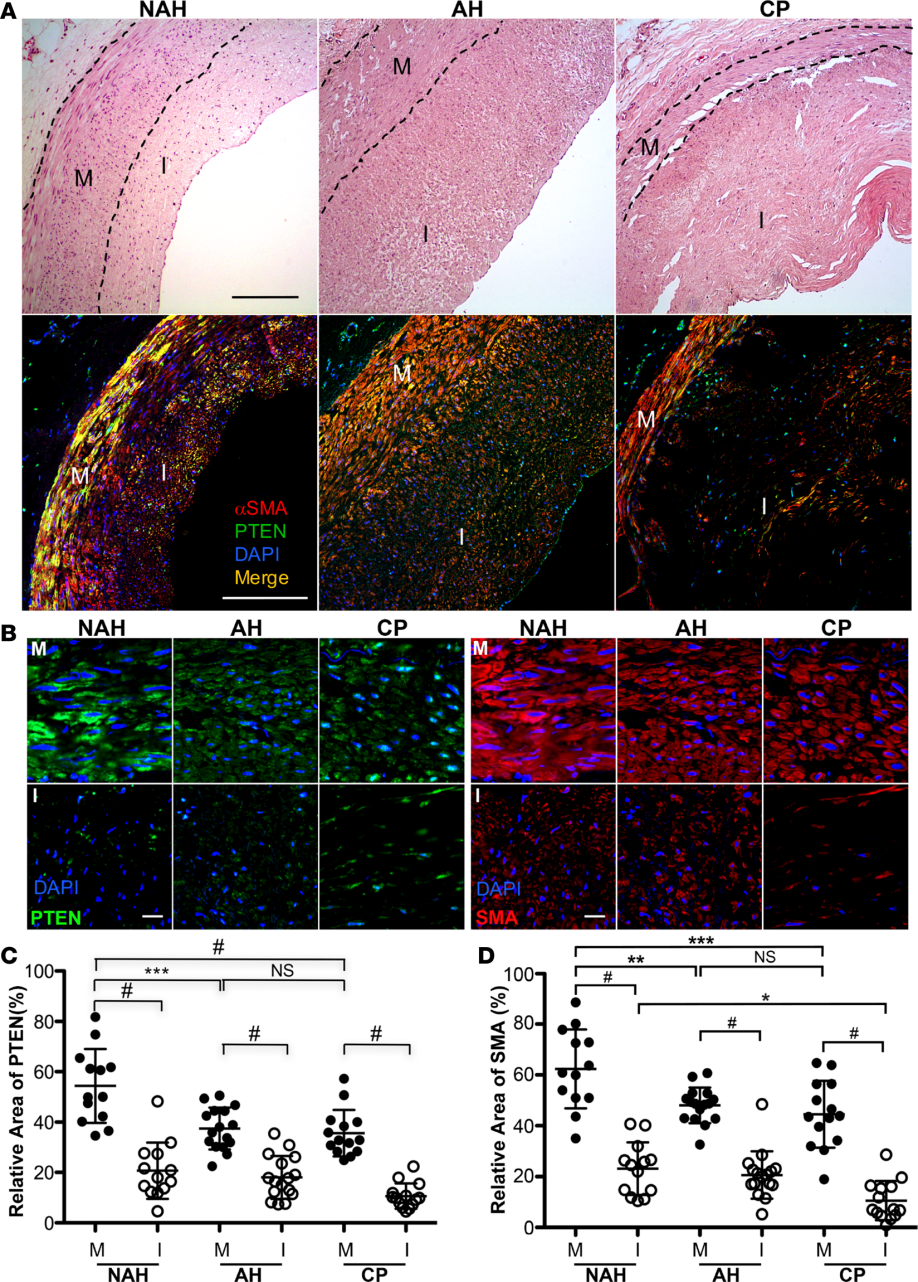
PTEN and α-smooth muscle actin (αSMA) expression in human atherosclerosis. (**A**) Hematoxylin and eosin (H&E; top panels) and PTEN (green) and αSMA (red) stained confocal images (bottom panels) of lesions representing nonatherosclerotic hyperplasia (NAH), atherosclerotic hyperplasia (AH; intima > 200 μm), or complex plaque (CP). Black dashed lines delineate the arterial media (M). I = arterial intima. Scale bars: 200 μm. (**B**) PTEN (green; left) and αSMA (red; right) stained confocal images of NAH (left in each group), AH (middle in each group), and CP (right in each group) lesions. PTEN and αSMA levels are more abundant in medial (M) smooth muscle cells (SMCs) compared with intimal (I) SMCs and are decreased in atherosclerotic lesions (AH, CP) compared with NAH lesions. Scale bars: 25 μm. (**C**) Relative area of PTEN expression in the arterial media (M) or intima (I) for a vessel lesion was measured by ImageJ and averaged from 4 or 5 confocal images (original magnification, ×63) of media and intima per vessel. (**D**) Relative area of αSMA staining was determined by ImageJ as described in **C**. NAH: *N* = 13 individual vessels from 7 independent hearts; AH: *N* = 16 individual vessels from 10 independent hearts; CP: *N* = 14 individual vessels from 11 independent hearts. Horizontal lines represent the mean ± SD. *P ≤ 0.05, **P ≤ 0.01, ***P ≤ 0.001, ^#^*P* ≤ 0.0001, by ANOVA with Bonferroni’s posttest multiple comparison test.

**Figure 3:**
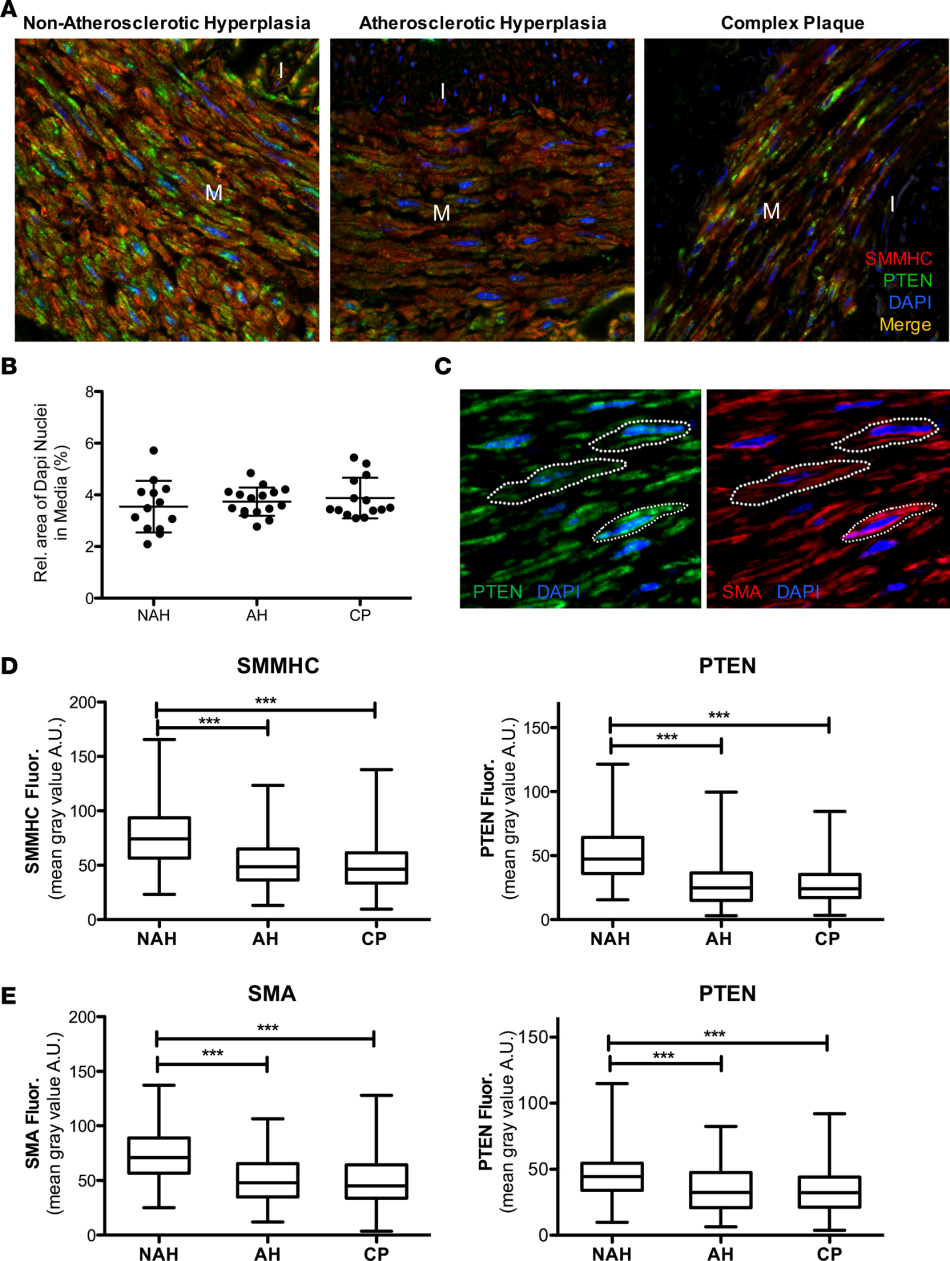
Single-cell analysis of PTEN and smooth muscle myosin heavy chain (SMMHC) α-smooth muscle actin (αSMA) expression in medial SMCs. (**A**) Representative PTEN (green) and SMMHC (red) stained confocal images of lesions representing nonatherosclerotic hyperplasia (NAH), atherosclerotic hyperplasia (AH; intima > 200 μm), or complex plaque (CP); merged images shown. M = arterial media; I = arterial intima. (**B**) Relative area of DAPI expression in the media per vessel was measured by ImageJ 1.47v as a measure of cellularity and averaged from 4 or 5 confocal images (original magnification, ×63) of media per vessel. NAH: *N* = 13 individual vessels from 7 independent hearts; AH: *N* = 16 individual vessels from 10 independent hearts; CP: *N* = 14 individual vessels from 11 independent hearts. (**C**) Representative confocal images of arterial media from NAH vessels immunofluorescently stained for PTEN (green; left) and αSMA (red; right); nuclei are stained with DAPI (blue). White dashed lines represent cell boundaries for ROI measurements. PTEN and SMMHC/αSMA levels within cell boundary ROI were measured by ImageJ 1.47v as described in Methods. (**D**) The mean gray value within cell boundary ROI for single-cell analysis of medial smooth muscle cells (SMCs) for SMMHC (left) and PTEN (right) levels was determined using ImageJ. NAH: *N* = 361 individual cells from 8 vessels and 5 independent hearts; AH: *N* = 294 individual cells from 7 vessels and 6 independent hearts; CP: *N* = 286 individual cells from 6 vessels and 5 independent hearts. ***P ≤ 0.001. Plotted data include the median gray value (horizontal bar), interquartile range (box boundary) and minimum to maximum range of data points (vertical bar). (**E**) The mean gray value within cell boundary ROI for single-cell analysis of medial SMCs for αSMA (left) and PTEN (right) levels was determined using ImageJ. NAH: *N* = 196 individual cells from 6 vessels and 6 independent hearts; AH: *N* = 197 individual cells from 6 vessels and 5 independent hearts; CP: *N* = 195 individual cells from 6 vessels and 5 independent hearts. ***P ≤ 0.001 by Kruskal Wallis with Dunn’s posttest comparisons.

To correlate loss of PTEN expression to loss of αSMA expression (as a conventional marker of differentiated SMCs) and severity of atherosclerosis, human coronary arteries were grouped based on histology into 3 categories from less disease involvement to severe disease involvement: NAH, AH (intima > 200 μm), and CP that are described in Methods and similar to 3 major classifications of coronary artery lesions previously described (Figure 2A). Confocal immunofluorescence imaging for PTEN and αSMA expression revealed that αSMA intensity was inversely correlated with disease progression and PTEN intensity correlated with αSMA levels (Figure 2A). Areas of PTEN and αSMA intensity in the vessel media and intima were quantified independently. For each vessel category, areas of intimal PTEN and αSMA expression were reduced compared with the media (Figure 2, B–D). Compared with NAH, PTEN and αSMA expression was reduced in the media of arteries with AH and CP (Figure 2, B–D). Compared with NAH and AH, αSMA expression was reduced in the intima of arteries with CP; differences in intimal PTEN expression among categories did not reach significance (Figure 2, B–D). A similar pattern was observed for SMMHC intensity (Figure 3A). Total cell number, as assessed by total DAPI intensity, was measured to determine if decreases in PTEN, αSMA, and SMMHC intensity in the media of AH and CP vessels was due to decreased cell number. Total medial SMC number was similar in all categories of diseased vessels (Figure 3B). Using single-cell analysis (Figure 3C), expression of PTEN, SMMHC, and αSMA was reduced in medial SMCs of arteries with AH and CP compared with NAH (Figure 3, D and E). Finally, to better control for individual patient differences, single-cell analysis of paired vessel segments with and without atherosclerotic plaque from the same artery of a small cohort of patients was performed. Compared with medial SMCs adjacent to uninvolved vessel areas, expression of PTEN and αSMA was reduced in medial SMCs adjacent to atherosclerotic plaques (Supplemental Figure 2). Collectively, the data support the concept that reduced SMC PTEN expression promotes SMC dedifferentiation and PTEN is depleted during atherosclerosis progression.

### SMC-specific PTEN deficiency accelerates atherosclerosis progression.

**Figure 4:**
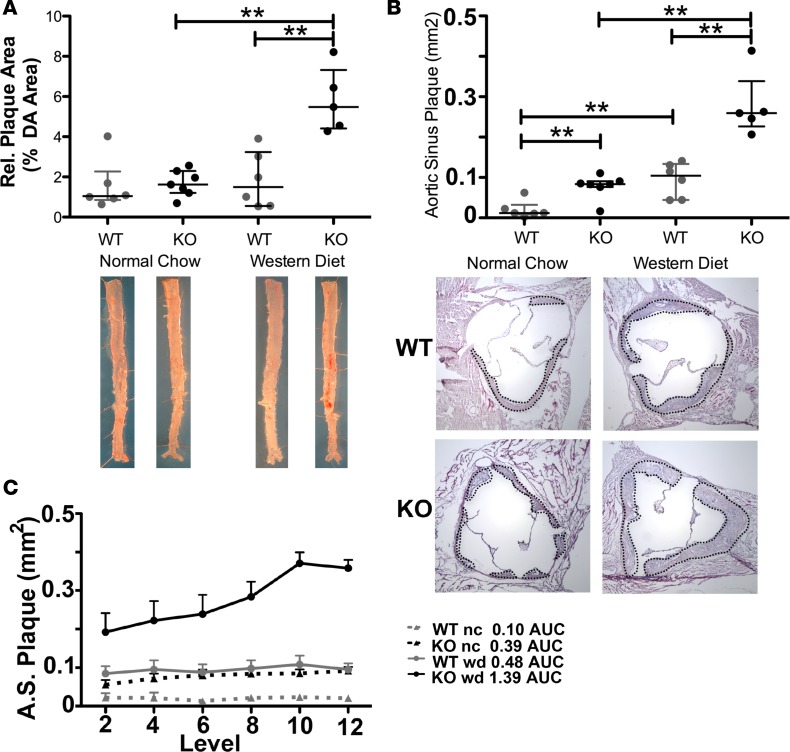
Accelerated atherosclerosis development in inducible, smooth muscle cell–specific PTEN-KO mice. *Myh11*-CreER^T2^; ApoE^–/–^ (WT) and PTEN-iKO; ApoE^–/–^ (KO) mice were injected with tamoxifen. Two weeks after the last tamoxifen injection mice were placed on normal chow (nc) or Western diet (0.15% cholesterol; wd) for 8 weeks. *N* = 6 WT nc, WT wd, KO nc; *N* = 5 KO wd. (**A**) The amount of atherosclerotic plaque in the aorta was measured as the percentage area of Sudan IV^+^ (red) plaques per area of en face–mounted descending aorta (DA). Representative aortas from each genotype and diet cohort are shown (bottom). (**B**) The area of intimal plaques was measured at 6 equally spaced levels in the aortic sinus and averaged. The mean area (mm^2^) of the aortic sinus plaque is plotted for each mouse. Representative H&E images of the aortic sinus plaques in WT and KO mice are shown for groups fed the normal chow or Western-type diet (bottom); black dashed lines outline area of plaque. (**C**) Aortic sinus (A.S.) plaque areas are plotted at each section level to perform area under the curve (AUC) analysis, as a correlate of plaque size. For all panels, gray data points indicate WT samples, black data points indicate KO samples, and horizontal lines indicate the median and interquartile range. **P < 0.01 by Kruskal Wallis with Dunn’s posttest comparisons.

Using inducible, SMC-specific PTEN-null mice (PTEN-iKO), we previously demonstrated that loss of SMC PTEN exacerbates injury-induced restenosis (33). To determine PTEN’s role in progression of primary atherosclerosis, PTEN-iKO mice were bred to ApoE-null mice. Wild-type (WT; i.e., ApoE^–/–^) and PTEN-iKO;ApoE^–/–^ mice were injected with tamoxifen to induce PTEN deletion (injections in WT mice served to control for any direct effects of tamoxifen) and then placed on normal chow or high-fat diet (Western type, high-fat diet containing 0.15% cholesterol) for 8 weeks. Plasma cholesterol and triglyceride levels and body weight following the 8-week diet are shown in Supplemental Table 6. While no differences were found between ApoE^–/–^ and PTEN-iKO;ApoE^–/–^ mice fed normal chow, PTEN-iKO;ApoE^-/-^ mice fed Western diet exhibited increased plasma cholesterol levels, but reduced plasma triglyceride levels and body weight compared with ApoE^–/–^ mice fed Western diet. By Sudan IV staining, the amount of atherosclerotic plaque in the descending aorta of PTEN-iKO;ApoE^–/–^ mice fed Western diet was considerably higher compared with plaque observed in WT mice on normal or Western diet as well as PTEN-iKO;ApoE^–/–^ mice fed normal chow (Figure 4A). Similarly, aortic sinus plaque area in PTEN-iKO;ApoE^–/–^ mice was considerably higher than plaque observed in the other 3 groups (Figure 4, B and C). Therefore, our mechanistic data in mice are in agreement with the human coronary artery findings that selective loss of PTEN in medial SMCs exacerbates atherosclerotic plaque progression.

### Medial remodeling and fibrosis in coronary arteries exposed to CF-LVAD correlate to decreased PTEN expression.

**Figure 5:**
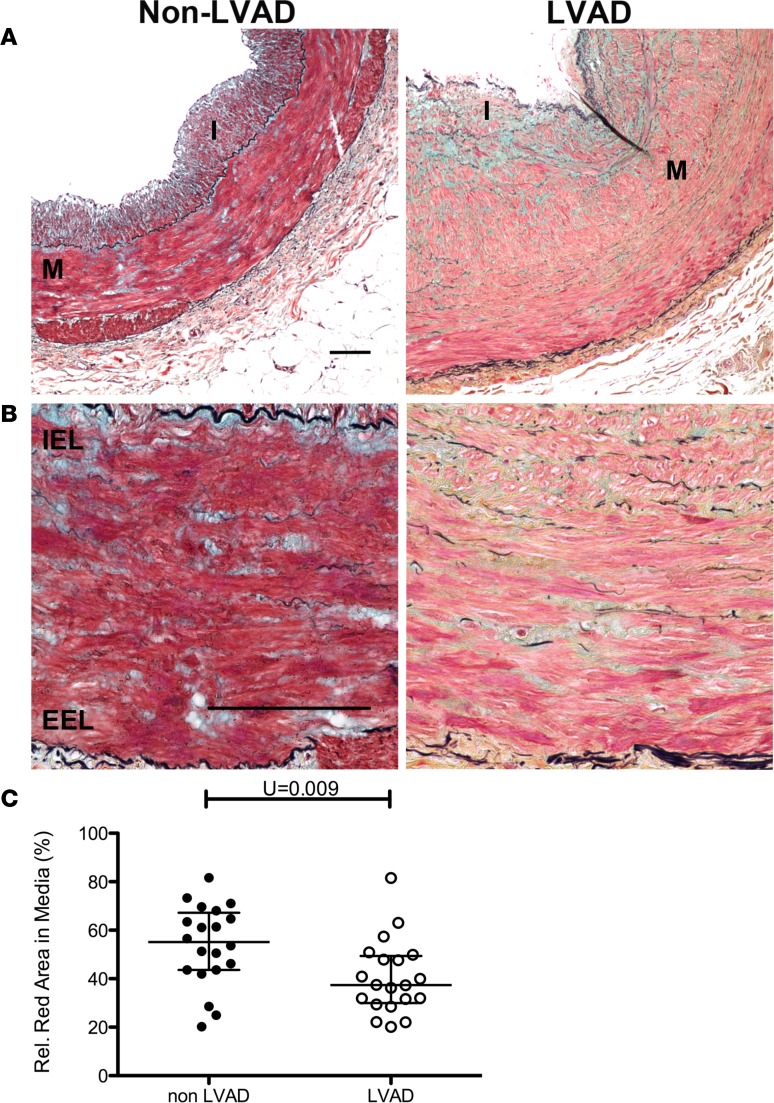
Smooth muscle dedifferentiation in coronary arteries exposed to CF-LVAD. Coronary arteries from explanted hearts of non– left ventricular assist device (non-LVAD) or continuous-flow LVAD (CF-LVAD) patients were stained with Movat’s pentachrome stain. (**A** and **B**) Representative low-power (**A**) and high-power (**B**) stained images showing decreased red staining in medial and intimal smooth muscle cells in CF-LVAD–exposed (right) compared with non–LVAD-exposed (left) vessels, indicative of decreased muscle. M = arterial media; I = arterial intima; IEL = internal elastic lamina; EEL = external elastic lamina.Scale bars: 100 μm. (**C**) The percentage red-stained area of the media was measured by ImageJ and averaged from 4 or 5 confocal images (original magnification, ×40) of media per vessel. Non-LVAD: *N* = 20 individual vessels from 11 independent hearts; CF-LVAD: *N* = 22 individual vessels from 12 independent hearts. **P = 0.009 by Mann-Whitney 2-tailed *U* test. Horizontal lines show the median and interquartile range.

**Figure 6:**
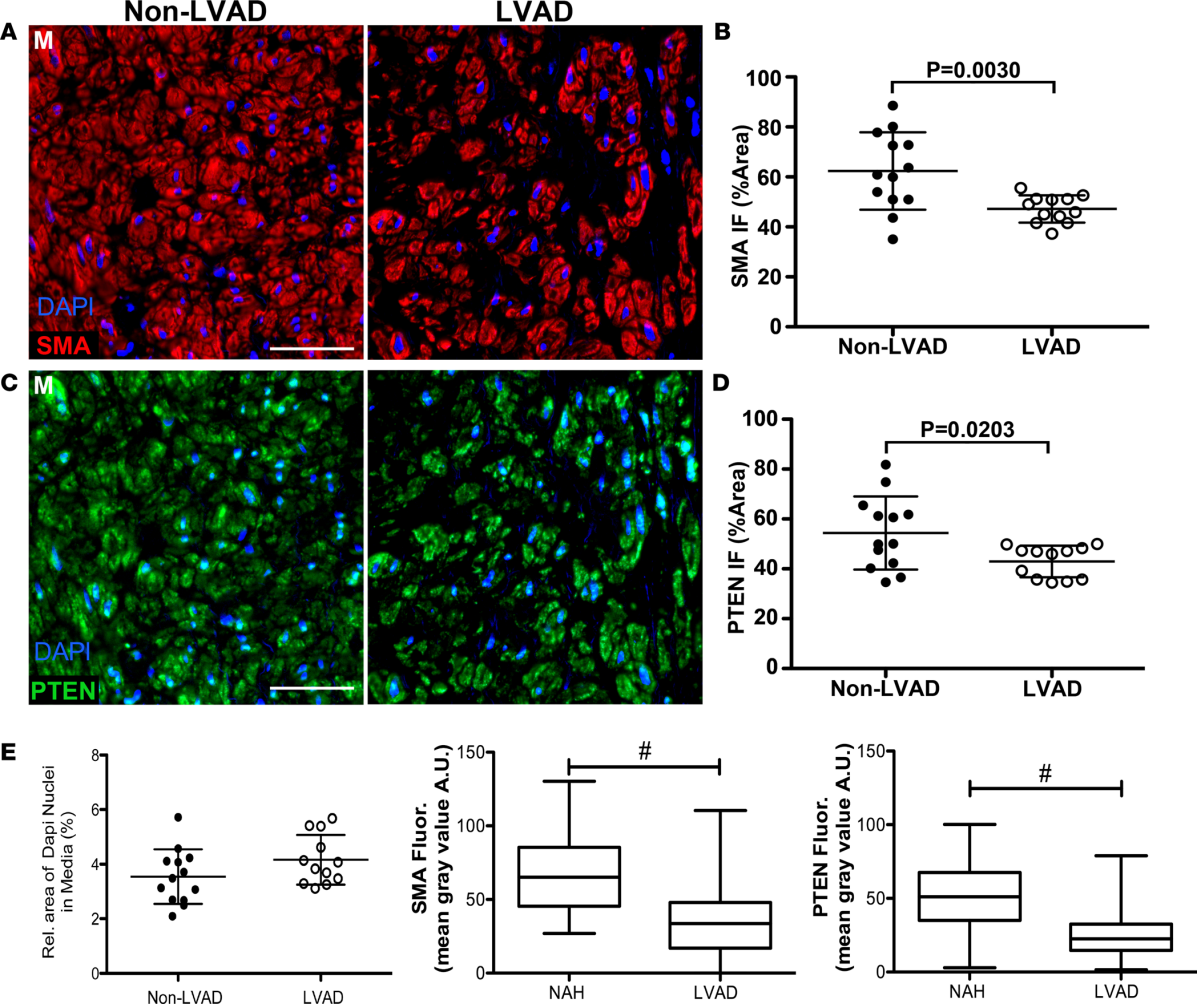
Decreased PTEN and αSMA expression in coronary arteries exposed to CF-LVADs. Coronary arteries from explanted hearts of non–left ventricular assist device (non-LVAD) or continuous-flow LVAD (CF-LVAD) patients were immunofluorescently stained for α-smooth muscle actin (αSMA) (**A**) and PTEN (**C**). (**A** and **C**) Representative confocal images of stained vessels showing the arterial media (M). Scale bars: 100 μm. (**B**) Relative area of αSMA expression for the arterial media was measured by ImageJ and averaged from 4 or 5 confocal images (original magnification, ×63) per vessel. (**D**) Relative area of PTEN staining was determined by ImageJ as described in **B**. Non-LVAD: *N* = 13 individual vessels from 7 independent hearts; CF-LVAD: *N* = 12 individual vessels from 9 independent hearts. Horizontal lines show the mean ± SD. Significant *P* values are shown, as determined by 2-tailed Student’s *t* test. (**E**) Left: Relative area of DAPI expression in the media per vessel was measured by ImageJ and averaged from 4 or 5 confocal images (original magnification, ×63) of media per vessel. Non-LVAD: *N* = 13 individual vessels; CF-LVAD: *N* = 12 individual vessels. Middle and Right: The mean gray value within cell boundary ROI for single-cell analysis of medial smooth muscle cells for αSMA (middle) and PTEN (right) levels was determined using ImageJ, as described in Figure 3. Nonatherosclerotic hyperplasia (NAH): *N* = 180 individual cells from 8 vessels and 5 independent hearts; LVAD: *N* = 217 individual cells from 7 vessels and 6 independent hearts. ^#^*P* ≤ 0.0001 by Mann-Whitney 2-tailed *t* test. Plotted data include the median gray value (horizontal bar), interquartile range (box boundary), and minimum to maximum range of data values (vertical bar).

**Figure 7:**
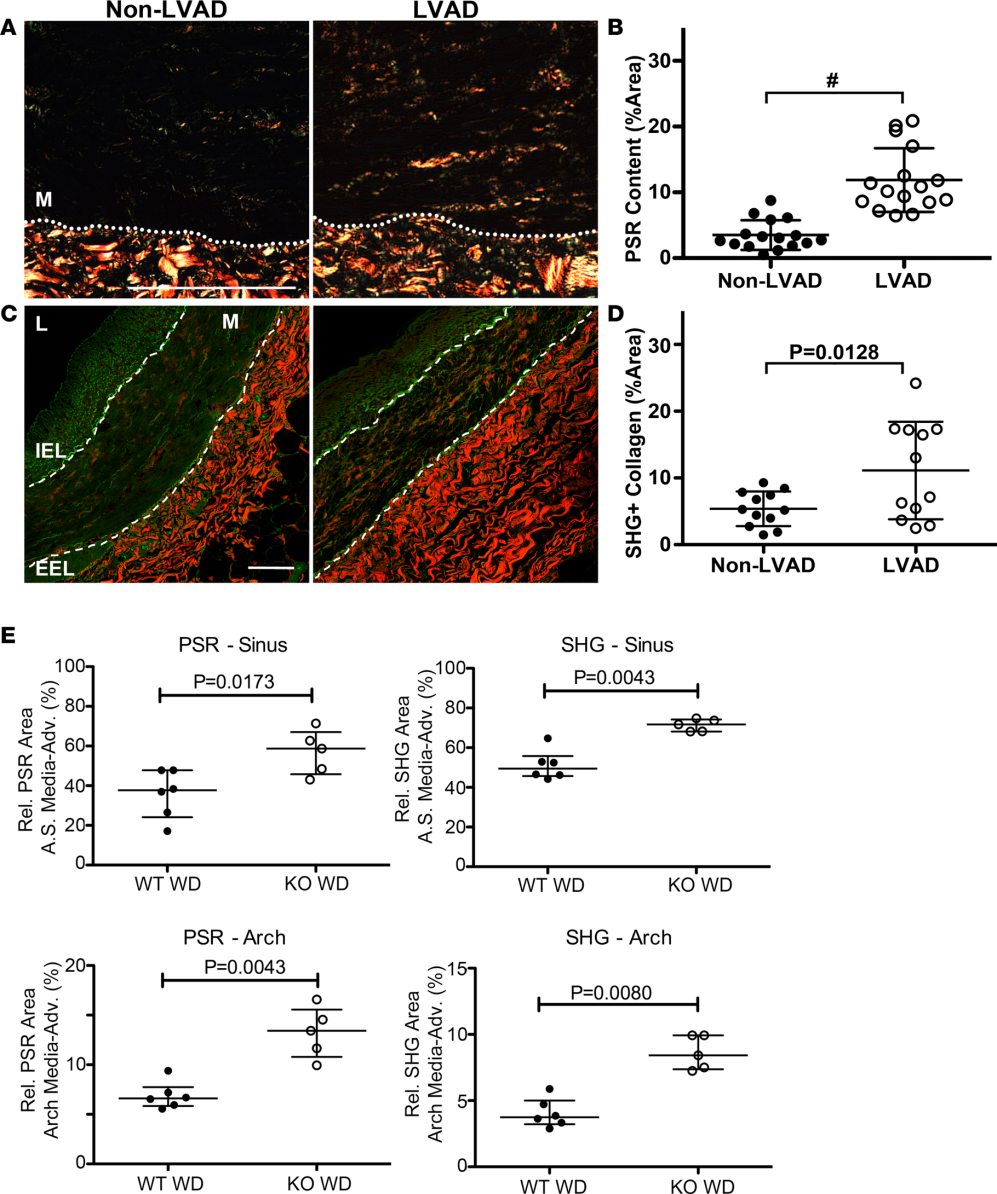
Increased collagen deposition in coronary arteries exposed to continuous-flow left ventricular assist devices (CF-LVADs). (**A**) Representative polarized light microscopy images of coronary arteries from explanted hearts of non-LVAD (left) or CF-LVAD (right) patients stained with picrosirius red (PSR) to quantify collagen deposition. Dashed lines separate the arterial media (M) from underlying adventitia. Scale bar: 100 μm. (**B**) Percentage PSR-positive area in the arterial media was measured by ImageJ and averaged from 4 or 5 images (original magnification, ×4) per vessel. non-LVAD: *N* = 16 individual vessels from 10 independent hearts; CF-LVAD: *N* = 16 individual vessels from 9 independent hearts. ^#^*P* ≤ 0.0001. (**C**) Representative images of coronary arteries from explanted hearts of non-LVAD (left) or CF-LVAD (right) patients imaged using label-free second harmonic generation confocal microscopy (SHG). Dashed lines delineate the arterial media. M, arterial media; IEL, internal elastic lamina; EEL, external elastic lamina. Scale bar: 100 μm. (**D**) Percentage SHG-positive area in the arterial media was measured by ImageJ and averaged from 4 or 5 images (original magnification, ×20) per vessel. non-LVAD: *N* = 12 individual vessels from 8 independent hearts; CF-LVAD: *N* = 12 individual vessels from 8 independent hearts. Horizontal lines indicate the mean ± SD. ^#^*P* ≤ 0.0001 by 2-tailed Student’s *t* test. (**E**) Sections of aortic sinus (top graphs) and aortic arch (bottom graphs) of tamoxifen-treated WT and PTEN-iKO mice placed on high-fat diet (WD), as described in Figure 4, were stained and imaged for PSR (left graphs) or processed and imaged for SHG microscopy (right graphs). Percentage PSR-positive or SHG-positive area in the combined media and adventitia was measured as described above. *N* = 6 WT WD, *N* = 5 KO WD. Horizontal lines show the median ± interquartile range. *P* values determined by 2-tailed Mann-Whitney *t* test.

Mechanical unloading of the heart with the use of CF-LVAD alters normal hemodynamic forces due to the nonphysiologic, nonpulsatile flow patterns. Our previous work demonstrated that aortas from CF-LVAD patients exhibited marked decreases in distensibility and increased vessel stiffness that was associated with increased collagen deposition (14, 15). Additionally, we showed that SMC-specific PTEN depletion in mice promotes spontaneous vascular fibrosis (31). In a separate study from the atherosclerosis analyses described above, using human NAH coronary arteries from non-LVAD compared with CF-LVAD patients, we examined the relationship between SMC PTEN expression and vessel structure and collagen deposition. NAH vessels were chosen for this study to eliminate the confounding effects of concurrent atherosclerosis on PTEN in order to determine whether PTEN is altered in arteries exposed to CF-LVAD and whether this corresponds to enhanced vascular remodeling. Arteries were matched for intimal hyperplasia (<250 μm; Supplemental Figure 3, A and B) and showed no difference in intima/lumen ratios between the non-LVAD and CF-LVAD coronaries (not shown). In addition, when normalized to lumen diameter to take into account vessels of differing size, no difference in medial thickness between non-LVAD and CF-LVAD arteries was observed (Supplemental Figure 3, A and B). Please note that while we observed dramatic changes in the adventitia of CF-LVAD arteries, these findings are the focus of another manuscript (AV Ambardekar, et al., under review). The present data related to CF-LVAD arteries is focused on the phenotypic changes of the media. Exposure to the altered hemodynamics produced by CF-LVAD was associated with SMC dedifferentiation, as assessed by reduced crocein scarlet–acid fuchsin staining for muscle (Figure 5) and by reduced αSMA levels (Figure 6, A and B). SMC dedifferentiation and reduced αSMA expression correlated with decreased SMC PTEN expression in arteries from CF-LVAD patients compared with non-LVAD patients (Figure 6, C and D). Total medial SMC number was similar in non-LVAD and CF-LVAD arteries (Figure 6E), indicating that reduced PTEN and αSMA intensity was not due to reduced SMC number in CF-LVAD arteries. To validate this we used single-cell analysis (as described in Figure 3), and compared with arteries from non-LVAD patients, expression of PTEN and αSMA was reduced in medial SMCs of arteries from CF-LVAD patients (Figure 6E). To determine if reductions in PTEN levels were associated with increased medial fibrosis, picrosirius red (PSR) staining combined with polarized light microscopy and second harmonic generation (SHG) microscopy were used to quantify collagen deposition. Increased PSR signal was detected in the media of CF-LVAD vessels compared with non-LVAD vessels, indicative of increased collagen deposition (Figure 7, A and B). Similar changes in collagen deposition were confirmed with SHG microscopy (Figure 7, C and D). Thus, altered hemodynamic forces on CF-LVAD arteries compared with non-LVAD arteries results in SMC PTEN deficiency that is associated with SMC dedifferentiation and vascular fibrosis. Finally, to mechanistically link loss of SMC PTEN to vascular fibrosis, aortic sinus and aortic arch sections from ApoE^–/–^ and PTEN-iKO;ApoE^–/–^ mice fed a high-fat diet for 8 weeks (as described in Figure 4) were stained for PSR or processed for SHG, as described above. Similar to human coronary arteries from CF-LVAD patients, increased collagen deposition was detected in the media of PTEN-iKO;ApoE^–/–^ vessels compared with ApoE^–/–^ vessels (Figure 7E), thereby confirming that loss of PTEN in SMCs promotes vascular fibrosis.

### PTEN-deficient SMCs exhibit a proinflammatory, profibrotic phenotype.

**Figure 8:**
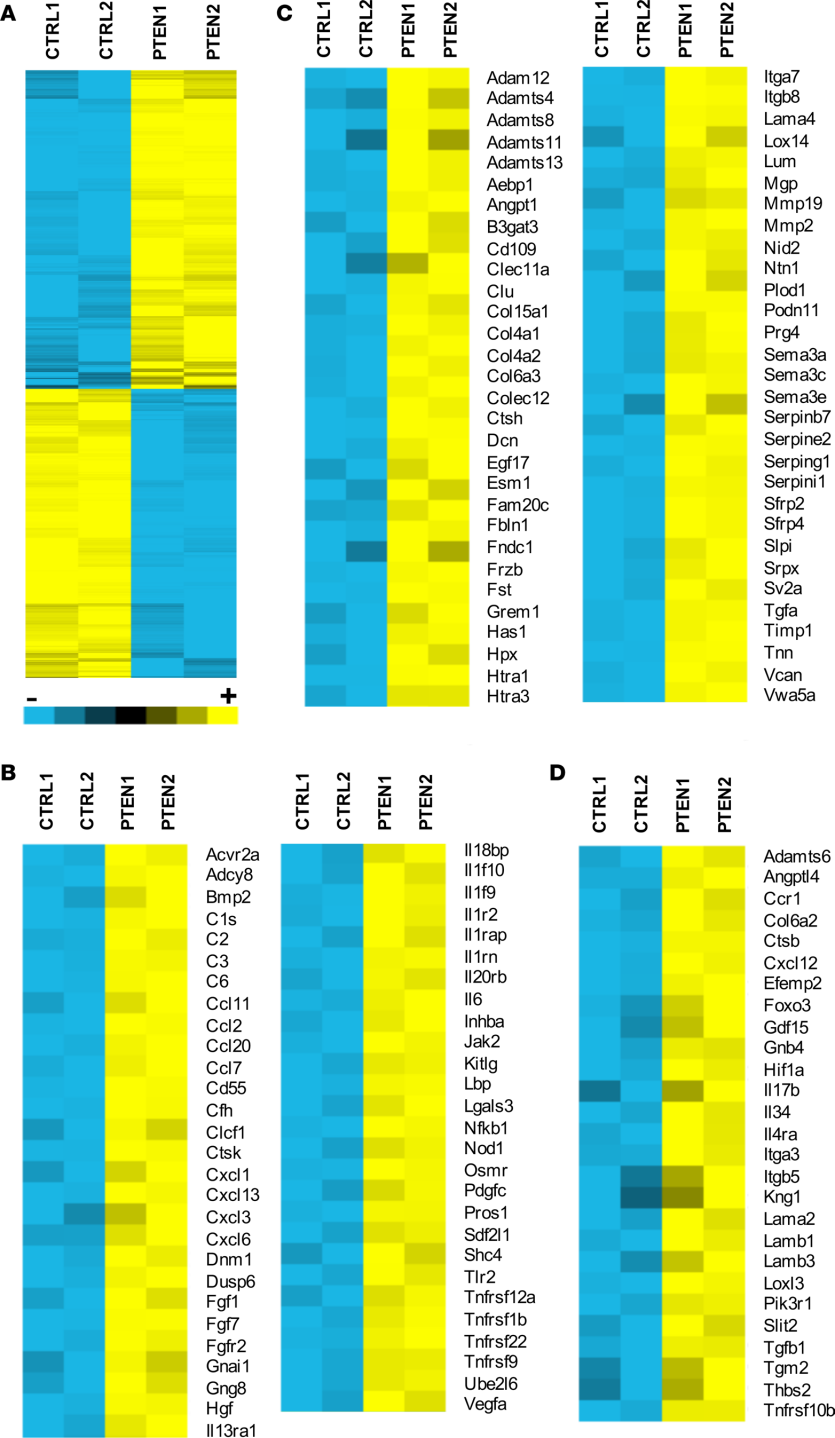
Loss of PTEN in smooth muscle cells (SMCs) promotes a proinflammatory, profibrotic phenotype. Control (CTRL1/2) or PTEN-deficient (PTEN1/2) SMCs were growth-arrested in media containing 0.1% calf serum for 48 hours. Total RNA was isolated from replicates and microarray analysis was performed using the Affymetrix RAE-230 rat Genechip. (**A**) Heatmap representing 1,173 differentially expressed genes. Yellow represents upregulated genes (591 genes upregulated in PTEN-deficient SMCs); blue represents downregulated genes (547 genes downregulated in PTEN-deficient SMCs). (**B**) The 1,173 differentially expressed genes were compared to previously published datasets of chemokine- and cytokine-associated genes. Heatmaps represent 55 genes that are upregulated in PTEN-deficient SMCs compared with control SMCs. (**C**) The 1,173 differentially expressed genes were compared to previously published datasets of extracellular matrix–associated (ECM-associated) and ECM remodeling–associated genes. Heatmaps represent 62 genes that are upregulated in PTEN-deficient SMCs compared with control SMCs. (**D**) Differentially expressed genes were compared to previously published cytokine and ECM datasets described above. Heatmap represents 27 genes that are upregulated 1.8- to 2.0-fold in PTEN-deficient SMCs compared with control SMCs.

**Figure 9:**
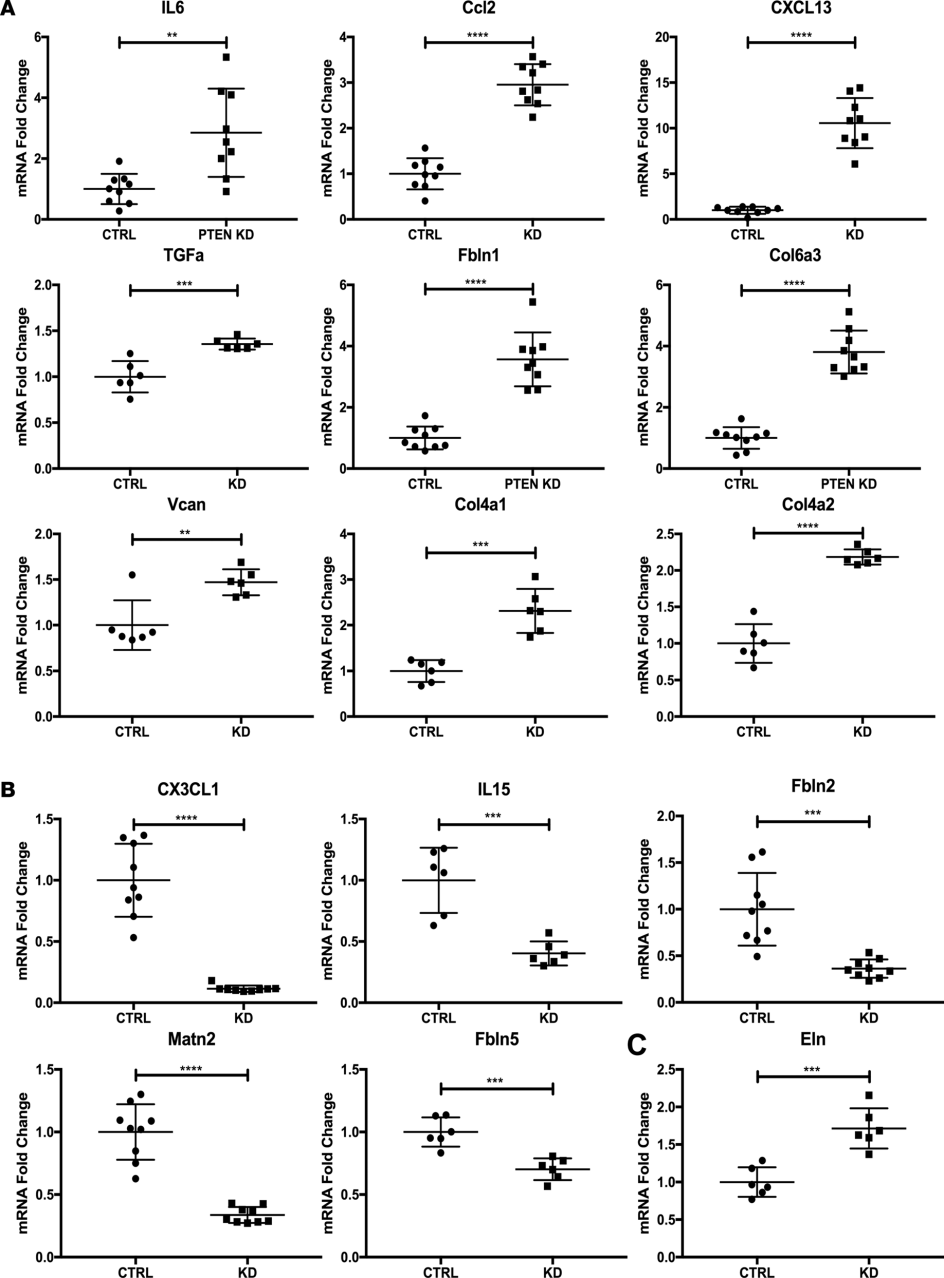
qPCR validation of gene changes in PTEN-deficient smooth muscle cells (SMCs) compared with control SMCs. Control (CTRL) or PTEN-deficient (PTEN KD) SMCs were growth-arrested in media containing 0.1% calf serum for 48 hours. Total RNA was isolated and quantitative RT-PCR (qPCR) was used to analyze for the indicated mRNAs. Horizontal lines indicate the mean ± SD. Shown are fold changes in mRNA copy number from triplicates or replicates of *N* = 3 independent experiments.**P < 0.01, ***P < 0.001, ****P < 0.0001 by 2-tailed Student’s *t* test. GAPDH was used for normalization. (**A**) Upregulated genes in PTEN-deficient SMCs compared with controls. (**B**) Downregulated genes in PTEN-deficient SMCs compared with controls. (**C**) mRNA that did not track with the microarray data.

To determine if loss of PTEN in SMCs alters global gene expression patterns related to inflammation and fibrosis, as observed in the setting of atherosclerotic arteries and in arteries exposed to CF-LVADs, control or PTEN-deficient SMCs were generated, as described previously (31–33). Microarray analysis was performed on control compared with PTEN-deficient SMCs using the Affymetrix Rat RAE-230 GeneChip, which contains 31,099 genes and expressed sequence tags. Samples were run in duplicate and our criteria for changes in gene expression were either a *P* value of 0.05 or a 2-fold change in value. Compared with controls, 1,173 genes were found to be differentially expressed (either increased or decreased expression) in PTEN-deficient SMCs (Figure 8A). We conducted pathway overrepresentation analysis of the differentially expressed genes using Web-based bioinformatic tools at ConsensusPathDB (http://cpdb.molgen.mpg.de/) (34, 35), searching in the KEGG and Reactome databases. Pathway overrepresentation analysis highly ranked multiple gene sets related to cytokine-cytokine receptor interaction and chemokine signaling as well as TGF-β signaling and ECM-related genes. The 1,173 differentially expressed genes were compared with previously published datasets related to cytokine/chemokine genes or ECM-related and ECM remodeling genes (36–39). Seventy-nine cytokine/chemokine–related genes were found to be common among our dataset and the existing datasets in the literature (Supplemental Figure 4). Of these, 55 genes were found to be increased, while 24 genes were found to be decreased in PTEN-deficient SMCs compared with controls (Figure 8B, Supplemental Tables 7 and 8). In addition, 118 ECM-related genes were found to be common among our dataset and the existing datasets in the literature (Supplemental Figure 5). Of these, 62 were found to be increased, while 56 were found to be decreased in PTEN-deficient SMCs compared with controls (Figure 8C and Supplemental Tables 9 and 10). In addition to these 2-fold or greater changes, an additional 27 cytokine/chemokine– or ECM-related genes were increased more than 1.8-fold in PTEN-deficient SMCs compared with controls (Figure 8D and Supplemental Table 11). In independent studies, we previously demonstrated that inactivation of PTEN resulted in a proinflammatory phenotype, as defined by increased expression of IL-6, MCP-1/CCL2, CXCL1, and SDF-1α/CXCL12 (31, 32). The microarray data are in agreement with these previous findings and extend these findings to demonstrate an overall global proinflammatory gene signature of PTEN-deficient SMCs. Using independent pools of PTEN-deficient SMCs, quantitative reverse transcription PCR (qPCR) for a subset of differentially expressed cytokine/chemokine and ECM-associated genes was used to further validate the microarray data. Supplemental Figure 6 shows loss of PTEN expression in PTEN-deficient SMCs, as measured by reduced total PTEN levels. In agreement with the microarray data, the cytokine/chemokine–related genes *Il6*, *Ccl2*, *Cxcl13*, and *Tgfa*, and the ECM-related mRNAs *Fbln1*, *Col6a3*, *Vcan*, *Col4a1*, and *Col4a2*, were found to be upregulated in PTEN-deficient SMCs compared with controls (Figure 9A). Similarly, *Cx3cl1*, *Il15* (previously demonstrated to have antifibrotic properties; see ref. 40), *Fbln2*, *Matn2*, and *Fbln5* were found to be downregulated in PTEN-deficient SMCs compared with controls (Figure 9B). *Eln*, shown as downregulated by microarray assessment, consistently was found upregulated in PTEN-deficient SMCs compared with controls using qPCR approaches (Figure 9C). Collectively, our data support the concept that PTEN deficiency promotes a proinflammatory and profibrotic SMC phenotype likely contributing to progression of atherosclerosis or pathological vascular fibrosis observed after exposure to CF-LVAD.

## Discussion

It is critical to validate that preclinical data generated from in vitro cell culture and in vivo animal studies is pertinent to human disease. A previous study used DNA microarray to identify differentially expressed genes in human atherosclerotic coronary arteries combined with meta-analysis to compare expression profiles of atherosclerotic coronaries to existing expression profiles from human atherosclerotic carotid arteries (41). PTEN was identified among the most commonly downregulated genes in both human coronary and carotid arteries. Our recent study showed that nuclear-localized and overall expression levels of PTEN were reduced in intimal SMCs of a small cohort of human atherosclerotic coronary arteries; however, the sample number was too small to draw conclusions on progressive loss of PTEN relative to the severity of atherosclerosis (28). Here, we extend our findings to establish that PTEN and SRF interact in the nucleus of differentiated SMCs in human vessels and that atherosclerosis progression involves an acquired deficiency of SMC PTEN with a secondary loss of SRF transcriptional activity, as defined by decreased SMC contractile protein expression. Mechanistically, this finding is supported by data showing accelerated progression of atherosclerosis in SMC-specific PTEN-null mice. Further, in a separate analysis, we demonstrated that vascular remodeling and fibrosis observed in nonatherosclerotic vessels of CF-LVAD patients are associated with overall reduced cellular content of PTEN and SMC dedifferentiation, which provides a novel mechanism involving PTEN for some of the altered vascular structure and functions reported in this patient population, in particular the impaired contractile properties, vasomotor regulation, and vascular fibrosis (14–18). Similar to the atherosclerosis studies and consistent with the findings in human coronary arteries exposed to CF-LVADs, SMC-specific PTEN-null mice exhibited increased susceptibility to vascular fibrosis. We previously reported that aortas of PTEN-deficient mice have impaired contractile properties (28). Importantly, PTEN inactivation results in downregulation of SMC-specific contractile genes (32) and a global gene expression signature that is proinflammatory and profibrotic, hallmarks of SMC dedifferentiation observed in diseased vessels.

Multiple lines of evidence have established that atherosclerosis is an inflammatory disease in which both the innate and adaptive immune systems participate in disease progression (2, 3, 9, 42, 43). Increasing information supports the concept that SMCs robustly respond to various stressors resulting in dedifferentiation and production of multiple cytokines and chemokines that affect macrophage and T cell function; SMC-derived cytokine and chemokine production is involved in all stages of atherogenesis (42, 43). Furthermore, the media is a site of relative immune privilege and vascular SMCs and their secreted ECM have the capacity to modify immune and other cell responses that manifest in the adjacent intimal and adventitial layers (9). As shown in our microarray data and our previous reports, SMC PTEN deficiency is associated with a global upregulation of proinflammatory genes, including *Cxcl12*, *Il6*, *Cxcl1*, *Ccl2*, and *Cxcl13* (Figure 9 and refs. 31, 32), and establishes that loss of PTEN promotes an inflammatory SMC phenotype leading to paracrine functions on immune cells. PTEN’s canonical function is dephosphorylation of PIP_3_, which inactivates the PI3-kinase/Akt pathway, and functions as a direct protein phosphatase (29, 30). Furthermore, our recent report described a novel, phosphatase-independent and SMC autonomous function of PTEN as a nuclear transcriptional cofactor with SRF that regulates SMC contractile gene expression (28). Collectively, PTEN regulates multiple pathways and functions that maintain vascular homeostasis; therefore, conditions that deplete its expression may contribute to the progression of atherosclerosis or other vascular diseases such as vasculitis or aneurysms. Thus, targeting PTEN represents a potentially novel and viable approach to reduce progression of disease.

Use of CF-LVADs continues to provide improved long-term survival of end-stage heart failure patients owing to the significant mechanical unloading that lowers left ventricular end-diastolic pressure (10). However, it is becoming increasingly recognized that exposure to nonphysiological, nonpulsatile blood flow in CF-LVAD patients leads to arterial wall thickness, increased collagen deposition, decreased compliance, and increased vessel stiffness (14–19). These structural and functional changes are likely major contributors to secondary adverse events observed in this patient population, such as hypertension, renal impairment, myocardial ischemia, and stroke. The current findings demonstrate a strong association between loss of SMC PTEN expression, SMC dedifferentiation, a shift towards a global profibrotic gene expression signature in PTEN-deficient SMCs, and pathological vascular fibrosis in arteries from CF-LVAD patients that mirrors the alterations of aortas in SMC-specific PTEN-null mice. Remodeling of coronary arteries in the setting of CF-LVAD has important clinical implications related to overall arterial remodeling. We and others have reported similar structural and functional changes in the aortas of CF-LVAD patients (14–17), while other studies have observed similar renal artery remodeling and peripheral vascular dysfunction after CF-LVAD support (18, 19). Clinically, fibrotic remodeling of the aorta and major artery branches could increase systemic vascular resistance and promote a chronic hypertensive state that persists after a subsequent heart transplant and could lead to end-organ damage and failure after heart transplant.

Our findings in coronary arteries from CF-LVAD patients imply the importance of physiological pulsatile blood flow in normal vascular homeostasis and propose a mechanism, namely pulsatile flow–mediated maintenance of PTEN expression. One limitation of this study was the small number of coronary arteries from patients implanted with new LVAD designs to determine whether these modified hemodynamic profiles will reduce the vascular remodeling and altered PTEN expression observed in the CF-LVAD–exposed arteries. While our study focused on SMC PTEN and resultant arterial medial changes, we propose that the SMC phenotypic changes observed in the media of CF-LVAD patients could significantly modify the structure of the adventitia (Ambardekar, et al., unpublished data), similar to the adventitial fibrotic responses observed in SMC-specific PTEN-null mice (31). Since human coronary arteries cannot be sampled at different times during the progression of atherosclerotic disease or before and after CF-LVAD implantation, this study is limited to assessing the temporal changes in PTEN expression. In addition, for the LVAD studies, we analyzed nonatherosclerotic human arteries to assess the influence of CF-LVAD exposure alone without the confounding effects of atherosclerosis. However, it would be interesting to examine the effects of combined atherosclerosis and CF-LVAD exposure in future studies to determine if concurrent atherosclerotic burden is worsened in association with CF-LVAD exposure.

Common features contributing to pathological vascular remodeling in the setting of atherosclerosis or CF-LVAD implantation include SMC dedifferentiation, inflammatory signaling and immune cell recruitment, and vascular fibrosis. Therefore, novel strategies aimed at reducing these events could be effective in preventing atherosclerosis progression and vascular structural and functional alterations observed in CF-LVAD patients. Indeed, the recent report from the CANTOS Clinical Trial supports the use of novel antiinflammatory approaches to protect against recurrent myocardial infarctions or stroke (44). Our data highly support a role for PTEN in human vessels as an antiinflammatory and antifibrotic target that functions to maintain the SMC differentiated phenotype. Interestingly, recent unpublished data from our lab demonstrate that systemic PTEN overexpression in mice blocks angiotensin-mediated cardiovascular fibrosis and accumulation of immune cells (Lu, et al., unpublished data) as well as atherosclerosis progression (Moulton, et al., unpublished data), further supporting this proposal. Moreover, previous studies examining the tumor suppressive functions of PTEN demonstrated that systemic elevation of PTEN results in a healthy and tumor suppressive anti-Warburg phenotype, reduced fat accumulation, and increased mitochondrial oxidative phosphorylation (45, 46). Collectively, these findings support the concept that systemic pharmacological upregulation of PTEN is a potentially novel and viable approach to prevent the detrimental structural and functional vascular changes associated with atherosclerosis or as observed in patients supported with CF-LVADs.

## Methods

### Human tissues

Human coronary arteries were obtained from explanted hearts of individuals undergoing cardiac transplantation for heart failure at the University of Colorado Anschutz Medical Campus and from nonfailing donor hearts that were not utilized for transplant for non-cardiac reasons. Additional deidentified clinical variables were recorded for each individual, including the age, gender, the cardiac diagnosis or type of cardiomyopathy leading to heart transplant, and the presence or absence of an LVAD. The Colorado Multicenter Institutional Review Board approved the protocol for the collection, storage, and analysis of human tissue (COMIRB 01-568).

Nonidentifying clinical variables of patients are shown in Table 1. A total of 87 human coronary arteries were obtained from 44 individuals with heart failure undergoing cardiac transplantation and from 5 nontransplanted hearts. Proximal right coronary, left main, left anterior descending, and left circumflex arteries with their surrounding epicardial fat were partially dissected from myocardial tissue and fixed overnight with 4% paraformaldehyde (PFA). Vessels with extensive calcification were placed in rapid bone decalcifier solution (American Master Tech) until they were sufficiently pliable to be cut. Vessel segments were processed for paraffin embedding and oriented to yield transverse artery sections. After histological processing and hematoxylin and eosin (H&E) staining, vascular sections were reviewed by K.S. Moulton and selected for this study into 3 groups (NAH, AH, or CP), which are groups based on a published classification of intimal hyperplastic lesions affecting human coronary arteries (47). NAH coronary artery segments had nonpathologic intimal thickening that contained SMCs and matrix without inflammatory and lipid deposits. AH coronary segments consisted of pathologic intimal thickening that contained SMCs and macrophages, but no lipid pools. Additionally, we limited our selection criterion to lesions with intimal thickening less than 250 μm. Coronaries classified as CP contained macrophages, lipid deposits, and fibrous caps (47). Coronary arteries with only NAH were selected from subjects with CF-LVADs and matched for intimal and medial thickness and intima/lumen ratios to NAH samples from non-LVAD patients in order to reduce the confounding effects of coexisting atherosclerotic lesions on vascular remodeling changes in coronaries arteries with and without exposure to CF-LVAD support.

### Animals

#### Inducible SMC-specific PTEN-iKO mice and atherosclerosis progression.

PTEN^fl/fl^ mice (Tak Mak, Ontario Cancer Institute, University of Toronto, Toronto, Ontario, Canada) and SMMHC (*Myh11*)-CreER^T2^ transgenic mice (Stephen Offermanns, University of Heidelberg, Heidelberg, Germany) were bred to generate tamoxifen-inducible SMC-specific PTEN-knockout mice (PTEN iKO) in the C57BL6/J genetic background, as described by us previously (33). Controls expressed *Myh11*-CreER^T2^ but were WT for PTEN. All mice were previously backcrossed more than 10 generations to the C57BL/6 background. Mice were maintained in the Center for Comparative Medicine, and procedures were performed under a protocol approved by the Institutional Animal Care and Use Committee at the University of Colorado Denver. Only male mice were used, as the *Myh11*-CreER^T2^ BAC transgene inserted on the Y chromosome. PTEN-iKO mice were bred to ApoE^–/–^ mice (stock 007069; Jackson Laboratories) to generate PTEN-iKO;ApoE^–/–^double-knockout mice. ApoE^–/–^ mice were used as controls (*Myh11*-CreER^T2^; ApoE^–/–^). Mice (8–9 weeks old) received 1 μg tamoxifen injections i.p. for 5 consecutive days to induce PTEN knockout. Two weeks following the first tamoxifen injection (as described above), ApoE^–/–^ or PTEN-iKO;ApoE^–/–^ mice were provided normal chow or Western diet containing 0.15% cholesterol (42% calories from fat; Western type 88137, Teklad) for an additional 7 or 8 weeks. Following diet, mice were anesthetized, serum was obtained for cholesterol and triglyceride measurements, and mice were perfused with 2% PFA. Hearts, whole aortas, and carotid arteries were dissected and placed in 2% PFA. Whole aortas were stained with Sudan IV and pinned open en face to measure the percentage area of atherosclerotic plaques that involved the descending aorta. Hearts were cut along the plane of the AV groove and embedded in OCT to collect serial transverse sections (8 μm) of the aortic sinus along a 700-μm distance. The transverse areas of intimal plaques (mm^2^) were measured at 6 equally spaced intervals and averaged for each mouse. In addition, the aortic sinus plaque areas were plotted for each section level to perform area under the curve (AUC) analysis, as a correlate of plaque size. Serum cholesterol and triglyceride levels were measured relative to standard control samples by automated colorimetric assays (Wako Chemicals). For fibrosis quantification, the aortic sinus and aortic arch were imaged by SHG microscopy or stained with PSR and imaged using polarized light microscopy (see below).

### PLA

Formalin-fixed, paraffin-embedded (FFPE) tissues were deparaffinized and rehydrated, rinsed in PBS, and underwent antigen retrieval by heating for 10 minutes at 100°C in a decloaking chamber (Biocare). After cooling they were blocked for 1 hour in TNB blocking solution and incubated at 4°C overnight with mouse monoclonal anti-PTEN (A2b1) antibody (1:50; Abcam) and rabbit monoclonal anti-SRF (H-300) antibody (1:200; Santa Cruz Biotechnology). PTEN-SRF interaction was determined using a DUOLink PLA Kit according to the manufacturer’s instructions using DUOlink PLA Probe anti-Mouse PLUS, DUOlink PLA Probe anti-Rabbit MINUS, and DUOlink In Situ Detection Reagents Orange (All DUOlink reagents obtained from Sigma-Aldrich). Positive controls for SRF and PTEN alone used anti-Rabbit PLUS and anti-Rabbit MINUS (SRF) and anti-Mouse PLUS and anti-MOUSE MINUS (PTEN). Negative controls omitted the respective SRF or PTEN primary antibody. A specificity control for the PLA signal for SRF-PTEN interactions was demonstrated by loss of the PLA signal when either the SRF or the PTEN primary antibody was omitted. Slides were mounted with DUOLink In Situ Mounting Medium with DAPI (Sigma-Aldrich). Sections were visualized with a laser-scanning confocal microscope (Zeiss LSM 780) and ImageJ 1.47v software (NIH).

### Immunofluorescence and confocal microscopy

FFPE coronary arterial tissues were deparaffinized, rehydrated, and underwent antigen retrieval as described above. Tissues were stained with H&E or Movat’s pentachrome for vessel morphology, composition, and measurements of intima and media dimensions. For immunofluorescent double staining for PTEN and αSMA or SMMHC, tissues were labeled by an overnight incubation with a monoclonal (clone Y184) rabbit anti-PTEN antibody (1:50; catalog 04-409, Millipore) and a Cy3-conjugated monoclonal (clone 1A4) mouse anti-αSMA antibody (1:1,000; catalog C6198, Sigma-Aldrich) or mouse anti-SMMHC antibody (1:100; catalog ab81031, Abcam). Following incubations with the primary antibodies, PTEN was detected after sequential incubations with secondary biotinylated goat anti-rabbit IgG (1:500; catalog BA-1000, Vector Laboratories) and steps detailed in the TSA Biotin amplification method (catalog NEL700A001KT, Perkin Elmer), including streptavidin conjugated-HRP (1:100), biotinyl tyramide (1:50), and Alexa-488–conjugated Streptavidin (1:200, catalog S11223, Thermo Fisher Scientific). An Alexa-568–conjugated goat anti-mouse IgG (1:500; Invitrogen) was used to detect αSMA or SMMHC. Sections were mounted in Vectashield media containing DAPI (catalog H-1200, Vector Laboratories) to label cell nuclei. All images of PTEN and αSMA or SMMHC fluorescence were obtained using a Zeiss 780 laser-scanning confocal microscope and Zen software (Carl Zeiss) set at the same configuration for all slides and for individual antigens within a 4-μm optical plane of the tissue section, thereby capturing intensity from a larger volume of SMCs that are positioned in this optical plane.

### Image analysis

The areas of positive PTEN and αSMA fluorescence relative to the area of the vessel media and intima were quantified independently with ImageJ 1.47v. The media area was measured between the internal and external elastic laminae borders; intima area was measured between the arterial lumen and internal elastic lamina. The cell density of vessel media was measured by determining the area of nuclear DAPI fluorescence using ImageJ 1.47v.

Single-cell analysis of the abundances (mean gray value) of PTEN and αSMA or PTEN and SMMHC in individual medial SMCs was quantified using ImageJ 1.47v by 2 independent observers. Each artery sample was imaged with 4 or 5 nonoverlapping images at ×63 magnification and the same confocal configurations. Individual SMCs in the media were selected for analysis if the SMC had discernible cell borders and a confluent nucleus, which indicated the selected cells were positioned with a significant cellular volume residing within the 4-μm optical imaging plane, which also approximated the thickness of SMC nuclei. Based on this cell-selection criterion, the PTEN and SMC fluorescence intensities were sampled from the mid-section of aligned cells within the media and not the edge of an SMC. A range of 7–10 SMCs were analyzed from each image and cell boundary ROIs were drawn around each SMC to measure the mean gray value of PTEN and αSMA or SMMHC immunofluorescence for each cell. Cell-wise intensity values of PTEN and αSMA or PTEN and SMMHC intensities were collected on a minimum of 195 cells for each comparison group (NAH, AH, and CP) and NAH non-LVAD versus NAH CF-LVAD.

### Histology, Movat’s pentachrome Stain, PSR stain, SHG microscopy, and fibrosis quantification

Fibrosis was detected in the vessel media of tissue sections processed for Movat’s pentachrome staining, PSR, and SHG microscopy. Russell-Movat pentachrome staining was performed according to the manufacturer’s instructions (American MasterTech) on FFPE sections that were deparaffinized and rehydrated slides and stained with Verhoeff’s elastic stain, Alcian blue for mucin, crocein scarlet–acid fuchsin for muscle, and saffron solution for collagen. The slides were dehydrated and coverslips were mounted with VectaMount Permanent Mounting Medium (Vector). Images were visualized with an Olympus microscope and SPOT software. Percentage of red-stained area in the vessel media was quantified using ImageJ 1.47v. For PSR staining, FFPE tissues were deparaffinized in xylene and rehydrated in ethanol. They were incubated overnight at room temperature in Bouin’s solution (Sigma-Aldrich). The slides were then rinsed in running distilled water for 1 hour, stained in Picro-Sirius Red (American MasterTech) solution for 4 hours, washed in 2 changes of acidified water (0.5% glacial acetic acid in distilled water), and dehydrated in 100% ethanol and xylene. Coverslips were mounted with VectaMount Permanent Mounting Medium. Images were visualized with an Olympus microscope equipped with polarizing filters and SPOT software and all the images of samples in the experiment were collected at the same exposure settings. Medial fibrosis was quantified using polarized light microscopy and ImageJ 1.47v software. For label-free SHG microscopy, paraffin sections of artery segments were rehydrated and imaged via multiphoton excitation using a Zeiss LSM780 light microscope equipped with a femtosecond pulsed Ti Sapphire laser (Chameleon Ultra; Coherent) and ZEN software (2012 SP1 Black Edition). Images were acquired at 1,024 × 1,024 pixels (708.49 μm × 708.49 μm) using a Zeiss C-Apochromat ×20 objective. The excitation source was tuned for 800 nm to generate an SHG 400 nm signal that was collected via a 390–410 nm emission filter. Besides the SHG signal, the autofluorescence was also observed via a wide-range visible filter (420–700 nm). The area and percentage content of SHG^+^ collagen in the media were measured in 4 nonoverlapping ×20 images of the coronary artery. Cryosections of the aortic root and aortic arch collected from ApoE^–/–^ or PTEN-iKO;ApoE^–/–^ mice were processed for PSR staining and SHG microscopy as described above to compare for differences in fibrosis of the aorta.

### Cell culture, microarray analysis, and qPCR

Primary rat aortic SMCs were isolated and cultured as previously described (31). Briefly, the aggregate population of aortic medial SMCs from adult Sprague Dawley rats was aseptically dissected and SMCs were obtained by digestion in Eagle’s MEM medium (EMEM) containing collagenase and elastase. Isolated cells were maintained in EMEM containing 10% calf serum (CS) and were used as primary cultured cells through passage 10. PTEN-deficient rat aortic SMCs were generated as previously described (31–33). A short hairpin RNA (shRNA) sequence based on the mouse PTEN gene (NCBI accession NM008960) was designed and subcloned into a retroviral expression vector driven by the U6 promoter (Open Biosystems). To generate stable SMC clones, control or PTEN shRNA vectors were packaged into replication-defective retrovirus, as described previously. SMCs were incubated with secreted virus for 48 hours and cells expressing control or PTEN shRNA were selected by culturing in medium containing puromycin. Individual clones were screened by immunoblotting with total PTEN and phospho-Akt antibodies. Control or PTEN-deficient SMCs were cultured in EMEM supplemented with 0.1% CS for 48 hours and subjected to total RNA isolation by using RNeasy miniprep (Qiagen). Labeling of the probes for microarray was performed as described previously (48). Briefly, cDNA was synthesized by using a Superscript Choice System (Invitrogen), and purified by phenol/chloroform extraction and ethanol precipitation. In vitro transcription of labeled cRNA was performed from purified cDNA using a Bioarray high-yield RNA transcript labeling kit (Enzo Diagnostics) in the presence of biotinylated UTP and CTP. Labeled cRNA was purified and then fragmented using fragmentation buffer (40 mM Tris acetate, pH 8.1, 10 mM potassium acetate, and 30 mM magnesium acetate). Hybridization of the probes to the Affymetrix RAE-230 rat Genechip was preformed according to the manufacturer’s recommendations (Affymetrix) in the UCDAMC Microarray Core Facility. Intensity values were scaled and fluorescence intensity of each microarray was equivalent. DNA chips were scanned (6 μm resolution) with a HP Gene Array scanner. Significance analysis of microarrays (SAM) in the R Bioconductor package was used to select the list of differentially expressed genes at the *P* less than 0.05 cutoff value. Hierarchical clustering using Cluster 3.0 was performed with mean and normalized centering of the gene expression levels. Clustering was completed using centered correlation metric and complete linkage. Heat maps were generated using Java TreeView 1.1.6r4 and Matrix2png 1.2.1. The yellow, black, and blue color scale in Figure 8A represents the respective expression level of a gene above, equal to, or below the mean expression for that gene across all samples. Gene set comparisons were conducted against the KEGG, Reactome, and MSigDB databases (36–39). For qPCR to validate select genes demonstrating differential expression, pools of control or PTEN-deficient SMCs were generated with lentiviruses expressing control (nontargeting) or PTEN-specific shRNA (Sigma-Aldrich, TRCN0000322421), selected in puromycin, and expanded. SMCs used for qPCR validation were independent of the control and PTEN-deficient SMC clones used for microarray analysis. The shRNA targeting sequence 5′-CCGGCGACTTAGACTTGACCTATATCTCGAGATATAGGTCAAGTCTAAGTCGTTTTTG-3′ was used. SMCs were cultured in EMEM supplemented with 0.1% CS for 48 hours and total RNA was isolated by first digesting in RLT lysis buffer (Qiagen). Samples were then processed with QIAshredder and RNeasy Plus kits (Qiagen) to isolate RNA. First-strand cDNA was made using the iScript cDNA synthesis kit (Bio-Rad). Sequence-specific primers were designed for the following genes: *FBLN1* (fibulin 1), *Col4A1* (collagen type 4, α 1), *Col4A2* (collagen type IV, α 2), *Col6A3* (collagen type VI, α 3), *VCAN* (versican), and *IL15*. Primer sequences are available in Supplemental Table 1. qPCR was performed as previously described (32, 33) and GAPDH was used for normalization. Data were normalized to control shRNA SMCs (control SMCs set to “1” to average among independent experiments).

### Statistics

Data were analyzed using PRISM 5 (GraphPad Software, Inc.). Column statistics and D’Agostino and Pearson omnibus normality tests were performed to determine the mean, standard deviation, and normality of the data. For normal data, a 1-way ANOVA was used to determine if the overall *P* value was significant followed by Bonferroni’s multiple comparison to determine differences between the 3 (NAH, AH, and CP) groups. For non-normal data, a Kruskal-Wallis test was used to compare the 3 groups followed by Dunn’s multiple comparison tests. For comparisons between 2 groups (i.e., non-LVAD versus LVAD and mouse studies ApoE^–/–^ versus PTEN-iKO;ApoE^–/–^), unpaired, 2-tailed Student’s *t* tests for normal data and the Mann-Whitney test for non-normal data were used and reported with exact *P* values. A *P* value of 0.05 or less was considered significant.

### Study approval

Informed consent was obtained from patients prior to cardiac transplantation and human coronary arteries were collected under the Human Heart Tissue Bank protocol (Colorado Multiple Institutional Review Board Protocol 01-568) and coded with unique deidentified labels. Mice were maintained in the Center for Comparative Medicine, and procedures were performed under a protocol approved by the Institutional Animal Care and Use Committee at the University of Colorado Denver.

## Author contributions

MCMWE designed all the studies, analyzed and interpreted the data, and wrote the manuscript. KSM helped design experiments, obtained the human coronary artery specimens and categorized vessel sections, analyzed the data, performed image analysis, conducted the mouse atherosclerosis studies, and edited the manuscript. KS contributed to the cell culture qPCR studies. ML performed immunofluorescence studies of human coronary arteries and obtained images. SB performed the microarray analyses. PM performed single-cell analyses. RT maintained, bred, and genotyped the mouse lines, assisted with atherosclerosis studies, and performed qPCR studies. SBF analyzed the PSR stains. SL assisted with all mouse studies, processing of tissues, and generation of PTEN-deficient SMCs. BK assisted with single-cell analyses. JCC is the transplant surgeon responsible for human tissue collection. RAN assisted with the design of the studies and edited the manuscript. AVA is the co-Director of the Human Heart Tissue Bank and edited the manuscript.

## Supplementary Material

Supplemental data

## Figures and Tables

**Table 1:**
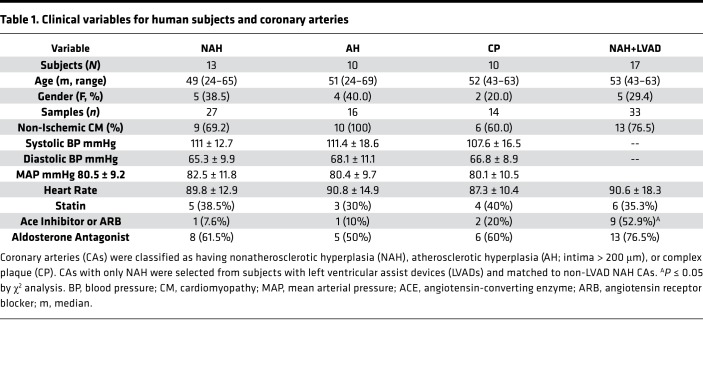
Clinical variables for human subjects and coronary arteries

## Version 1 02/22/2018

Electronic publication
