# Supplementary material for: PTEN deficiency promotes pathological vascular remodeling of human coronary arteries
Source: JCI Insight. 2018 Feb 22;3(4):e97228. doi: 10.1172/jci.insight.97228 (PMC5916252; doi:10.1172/jci.insight.97228)
Supplement: Supplemental data [file jciinsight-3-97228-s001.pdf]

Supplemental Table 1: Primer Sequences for qPCR

Gene Name	Forward Primer	Reverse Primer
Fbln1	GACTGCTCGCTCTCCTACAC	GCTCCTCCAGTTGGTTGTGA
Col6a3	GCCTGATTGGGGAACAAGGA	ATTTCCAGTGCCTCCCTTCG
Vcan	GATCATCTGGACGGCGATGT	GTTTGCACACTCTGCTTCGG
Col4a2	GGGAGAGGCTGGATTCTTCG	TCATCCCCGGCAGGATTAGA
Col4a1	CTCTGTTGGTCCCCCGC	CCAGAGCCACCACAATCACC
Il15	AGAGTTGGACGAAGAGGGGA	TGCTTTGAAGAGCCAGAGGG
Il6	CACTTCACAAGTCGGAGGCT	TCTGACAGTGCATCATCGCT
Ccl2	TGGGCCTGTTGTTCACAGTT	GAGTAGCAGCAGGTGAGTGG
Cxcl13	CACCTCCAGGCAGAATGAGG	TCGAGCTCACCTTGGAACAC
Tgfa	CTCTGCTAGCGCTGGGTATC	TGTGGGAATCTGGGCACTTG
Cx3cl1	CCAATCCCAGTGACCTTGCT	CTCGTCTCCAGGATGATGGC
Fbln2	AGCTTTGCCAGCATCTTTGC	GACTCTCGTGCAGTGTCCAA
Matn2	GACTGCACTGCTGGAGAGTT	GACACTTCGGGAGCTGTCAA
Fbln5	CCACGATTTCCAGGCCTCTT	TCGTCCACATCCACACACTG
Eln	GACTTCTGGGAGCGTTTGGA	CCACCTGGCCTTGAAGCATA

Supplemental Table 2: Non-atherosclerotic Hyperplasia Coronary Artery Samples

Identifier	Vessel	Age	Sex	Pathology	PTEN	αSMA	Movat's	PSR	SHG
A0998	LAD	63	M	Familial cardiomyopathy	Y	Y	N	N	N
A1004	LAD1	64	F	Ischemic cardiomyopathy	Y	Y	N	N	Y
	LAD2				Y	Y	Y	Y	N
	RCA2				Y	Y	Y	Y	Y
A1007	RCA2	24	M	Non-ischemic CM	Y	Y	N	N	N
	LAD				Y	Y	Y	Y	N
	LCX				Y	Y	Y	Y	Y
	RCA3				N	N	Y	N	N
	LAD2				N	N	Y	Y	Y
	RCA1				N	N	Y	Y	Y
A1010	LCX	45	M	Familial CM V-tach	Y	Y	Y	Y	Y
	RCA3				N	N	N	N	N
	RCA2				N	N	Y	N	N
A1016	LAD	51	F	ASD congenital HD	Y	Y	N	N	N
	LCX				Y	Y	Y	N	N
A1024	Lmain	58	F	Non-failing donor	Y	Y	Y	Y	N
A1035	LAD	47	M	Hypertrophic CM	Y	Y	Y	Y	Y
	mRCA				Y	Y	Y	Y	Y
A0997	mLCX	45	M	Non-ischemic CM	N	N	Y	Y	Y
A1001	RCA2	28	F	Congenital Epstein anomaly	N	N	Y	Y	Y
A1043	LAD	65	M	Non-ischemic CM	N	N	Y	N	N
A0937	LCA	43	M	Non-failing donor	N	N	Y	Y	N
A1044	mRCA	52	F	Non-failing donor	N	N	Y	Y	N
	pRCA				N	N	Y	N	N
	LCX				N	N	N	Y	N
	Lmain				N	N	N	N	Y
A1013	RCA	49	M	Non-ischemic CM	N	N	Y	Y	Y

Abbreviations for Supplemental Tables 1-4: LAD = left anterior descending coronary artery; RCA = right coronary artery; LCX = left circumflex artery; Lmain = proximal left coronary artery; pr = proximal region of vessel; m = mid region of vessel; di = distal region of vessel; CM = cardiomyopathy; V-tach = ventricular tachycardia; ASD = atrial septal defect; HD = heart defect; ARVC = arrythmogenic right ventricular cardiomyopathy; Movat's = Movat's pentachrome stain; PSR = picrosirius red stain; SHG = second harmonic generation.

**numbers following vessel name (e.g. RCA2) refer to non-contiguous segments of the same vessel from the same patient.

Supplemental Table 3: Atherosclerotic Hyperplasia Coronary Artery Samples

Identifier	Vessel	Age	Sex	Pathology	PTEN	αSMA	Movat's	PSR	SHG
A0976	RCA	59	F	Aneurysm, hypertension	Y	Y	N	N	N
A0990	LAD	61	M	ARVC	Y	Y	N	N	N
	RCA				Y	Y	N	N	N
A0998	Lmain	63	M	Non-ischemic CM	Y	Y	N	N	N
A1001	Lmain	28	F	Congenital Epstein anomaly	Y	Y	N	N	N
A1007	Lmain	24	M	Non-ischemic CM	Y	Y	N	N	N
	RCA				Y	Y	N	N	N
A1013	LCX	49	M	Non-ischemic CM	Y	Y	N	N	N
	RCA				Y	Y	N	N	N
A1016	pRCA	51	F	ASD congenital HD	Y	Y	N	N	N
A1017	diRCA	57	M	Adriamycin CM	Y	Y	N	N	N
A1024	pRCA	58	F	Non-failing donor	Y	Y	N	N	N
A1033	LCX	69	M	Hypertrophic CM	Y	Y	N	N	N
	Lmain				Y	Y	N	N	N
	mLAD				Y	Y	N	N	N
	RCA				Y	Y	N	N	N

Supplemental Table 4: Complex Plaque Coronary Artery Samples

Identifier	Vessel	Age	Sex	Pathology	PTEN	αSMA	Movat's	PSR	SHG
A0976	LCX	59	F	Aneurysm, hypertension	Y	Y	N	N	N
A0997	Lmain	45	M	Non-ischemic CM	Y	Y	N	N	N
	mLCX				Y	Y	N	N	N
	RCA				Y	Y	N	N	N
A0998	LAD	63	M	Non-ischemic CM	Y	Y	N	N	N
A1010	Lmain	45	M	Familial CM V-tach	Y	Y	N	N	N
	RCA				Y	Y	N	N	N
A1019	diLAD	57	M	Ischemic CM	Y	Y	N	N	N
A1024	mLCX	58	F	Non-failing donor	Y	Y	N	N	N
A1035	mLCX	47	M	Hypertrophic CM	Y	Y	N	N	N
A1036	Lmain	55	M	Non-ischemic CM-sarcoid	Y	Y	N	N	N
A0937	RCA	43	M	Non-failing donor	Y	Y	N	N	N
A0920	LCX1	60	M	Ischemic CM	Y	Y	N	N	N
	Lmain				Y	Y	N	N	N

Supplemental Table 5: LVAD Coronary Artery Samples

Identifier	Vessel	Age	Sex	Pathology	PTEN	αSMA	Movat's	PSR	SHG
A0921	RCA	31	M	Non-iscemic CM	Y	Y	N	N	N
A1012	LCX1	39	F	Non-ischemic CM	Y	Y	N	Y	Y
	RCA2				N	N	Y	N	N
	LAD3				N	N	Y	Y	Y
	LAD1				N	N	Y	Y	Y
	LCX2				N	N	Y	Y	N
A1020	RCA4	44	M	Non-ischemic CM	Y	Y	N	Y	Y
	mRCA2				N	N	Y	Y	N
	mRCA1				N	N	N	Y	Y
A1023	prRCA	26	M	Non-ischemic CM (viral)	Y	Y	Y	Y	Y
	mRCA4				N	N	Y	N	N
A1025	Lmain	38	M	Non-ischemic CM	Y	Y	Y	Y	Y
	prRCA				N	N	N	Y	N
	mRCA2				N	N	Y	N	N
	diRCA1				N	N	Y	Y	Y
A1032	RCA1	37	M	Non-ischemic CM	Y	Y	Y	Y	Y
	LCX				Y	Y	N	N	N
	RCA2				Y	Y	Y	N	N
	pRCA1				N	N	Y	N	N
A0917	RCA	27	M	Familial CM	Y	Y	N	N	N
	LAD				Y	Y	N	N	N
A1028	mRCA	49	M	Ischemic CM	Y	Y	N	Y	Y
A1037	mRCA	40	M	Non-ischemic CM	Y	Y	N	N	N
A0989	Lmain	57	F	Ischemic CM	N	N	Y	N	N
A1030	LCX	56	F	Non-ischemic CM-sarcoid	N	N	Y	N	N
A1005	RCA1	31	M	Non-ischemic CM	N	N	Y	Y	Y
A1045	LAD	56	F	Ischemic CM	N	N	Y	N	N
	LCX2				N	N	Y	Y	N
A0991	LCX	55	F	Ischemic CM	N	N	Y	N	N
	LAD				N	N	Y	N	N
A1051	pRCA2	66	M	Ischemic CM	N	N	Y	N	N
A1048	pRCA2	66	M	Non-ischemic CM	N	N	Y	N	N
A0926	RCA	30	M	Familial NI CM	N	N	N	Y	Y

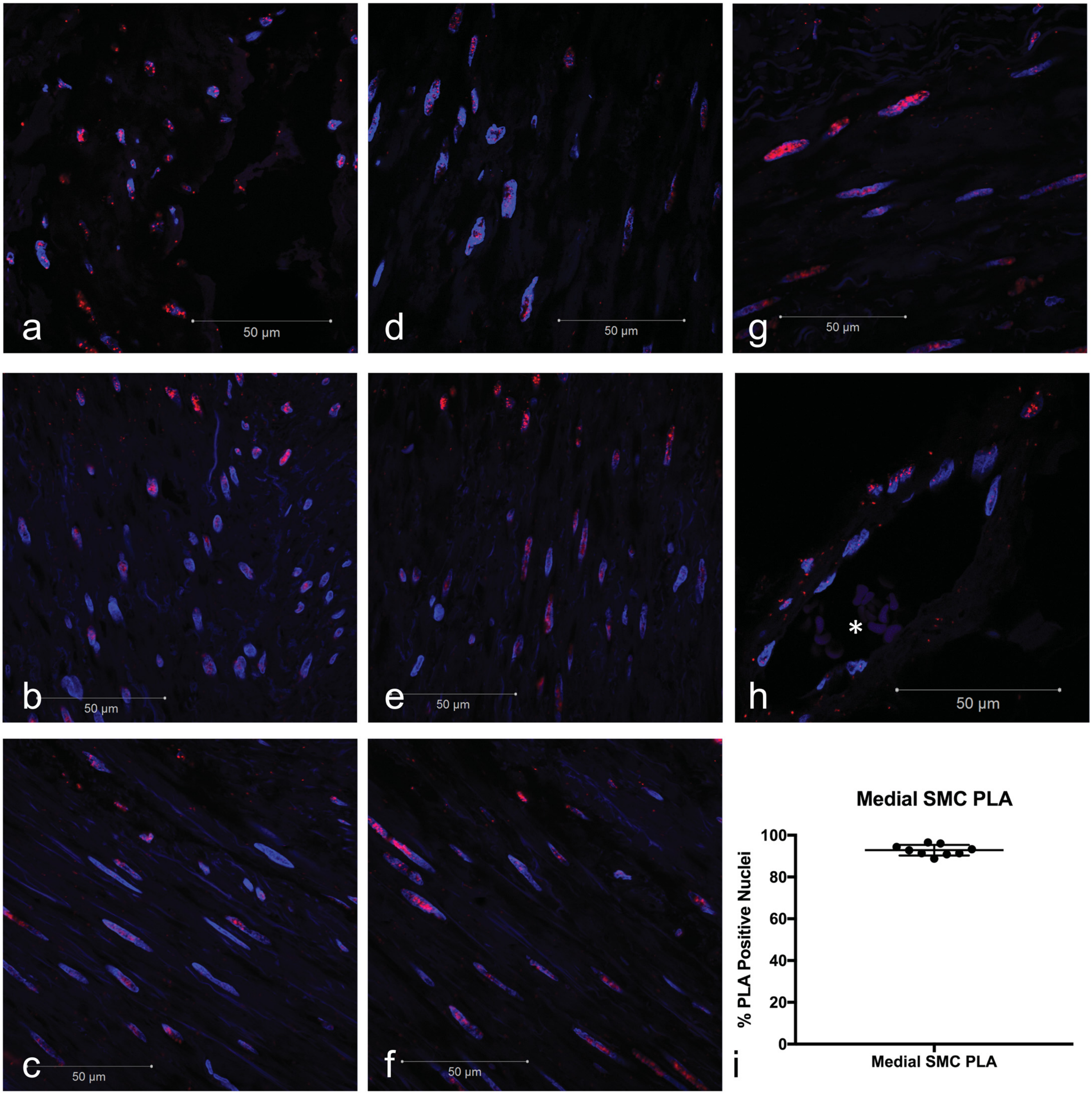

Supplemental Figure 1. Proximity Ligation Assay Showing Interaction of PTEN and Serum Response Factor (SRF) in the Nucleus of Medial Smooth Muscle Cells (SMCs). Proximity ligation assay (PLA) and confocal microscopy were used to detect PTEN-SRF interactions in medial SMCs of human non-atherosclerotic coronary arteries. **(a-g).** PLA using mouse anti-PTEN and rabbit anti-SRF primary antibodies and anti-mouse PLUS and anti-rabbit MINUS PLA probe demonstrates PTEN-SRF nuclear interactions in medial SMCs of human NAH coronary. Shown are several representative images from an N=4 vessels. **(h).** Positive PTEN-SRF PLA signal in SMCs of an adventitial vasa vasorum microvessel. * = red blood cells in lumen of microvessel. For panels a-h: Red = Positive PLA; Blue = DAPI for cell nuclei; Scale bars = 50 μm. **(i).** Nuclei of medial SMCs were scored for percent positive PLA signal. 9 representative images from N=4 individual non-atherosclerotic arteries from N=4 patients.

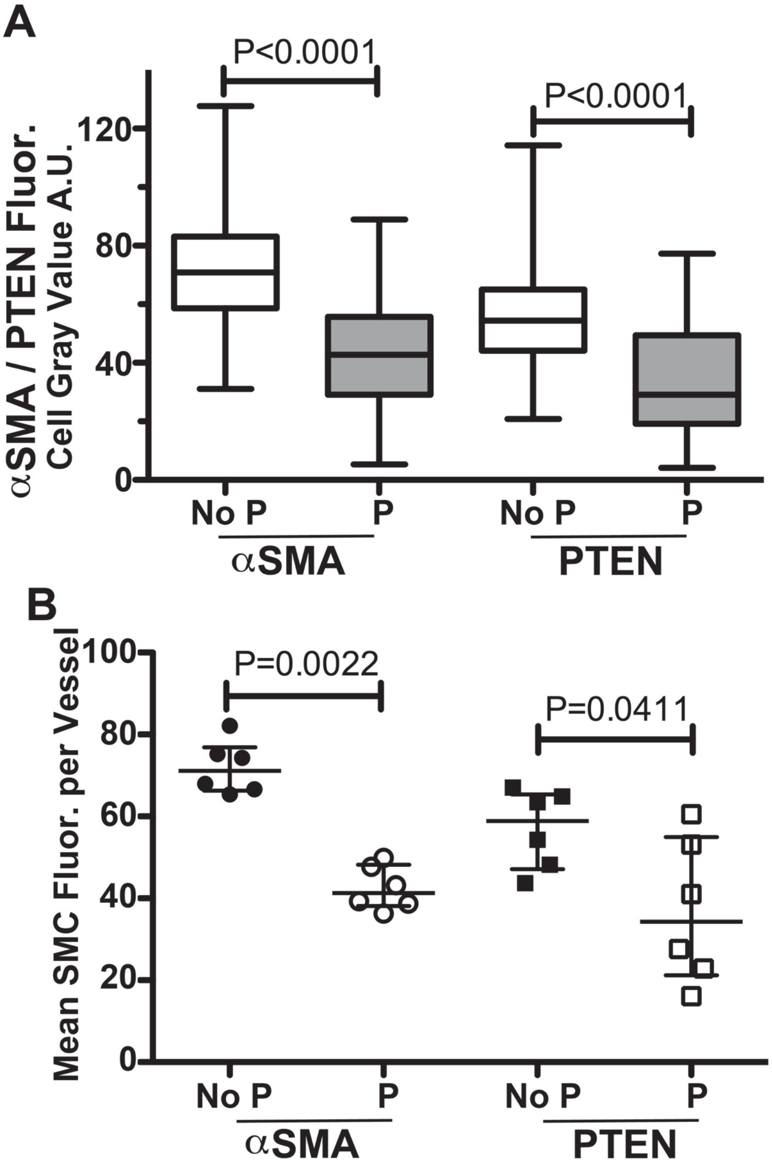

Supplemental Figure 2. Reduced PTEN and alpha-Smooth Muscle Actin (αSMA) in Medial Smooth Muscle Cells (SMCs) adjacent to atherosclerotic plaques. Paired vessel segments with and without atherosclerotic plaque (P, No P) from the same coronary artery were stained for PTEN and αSMA. Confocal microscopy was performed with the same image acquisition parameters used for single cell analysis of Figures 3 and 6. Four to five 63X images were acquired for each pair of atherosclerotic and non-atherosclerotic segments from the same coronary artery and independent heart. **A).** The mean gray values of αSMA and PTEN within the cell boundary were determined by Image J in 216 individual media cells of arteries with no plaque (open box) and 237 individual cells of media with adjacent atherosclerotic plaque (gray box) from N=6 vessels and independent hearts. Box and whisker data plots indicate the median gray value (bar), interquartile range (box boundary) and minimum to maximum range (error bars) of data values. **B)** The mean gray values for αSMA (circles) and PTEN (squares) of individual SMCs were averaged for each vessel (No P segment, filled; P segment, open). Exact P values are shown for Mann Whitney, two-tailed, t test comparisons of αSMA and PTEN gray values between coronary artery segments with and without adjacent plaque.

Supplemental Table 6: Mouse Plasma Cholesterol and Triglyceride Levels

	Normal Chow			Western Diet 0.15% cholesterol		
	WT	KO	t-test	WT	KO	t-test
Number	6	7		6	5	
Plasma Chol. (mg/dl)	100.2 (67.5-140.3)	119.5 (47.5-206.2)	0.7308	559.2 (513.6-593)	654.9 (614.5-703.9)	0.0173
Plasma TG (mg/dl)	175.7 (144.7-213.6)	136.7 (133.4-176)	0.0734	228.4 (182.2-257.4)	141.2 (114.1-188.5)	0.0303
Weight (gm)	29.4 (28.2-31.7)	30.5 (29.1-3.15)	0.6282	32.8 (31.3-34.4)	28.0 (27.3-30.2)	0.0173

All variables are median values with (interquartile range)

Mann Whitney t-test of WT versus KO for each diet condition.

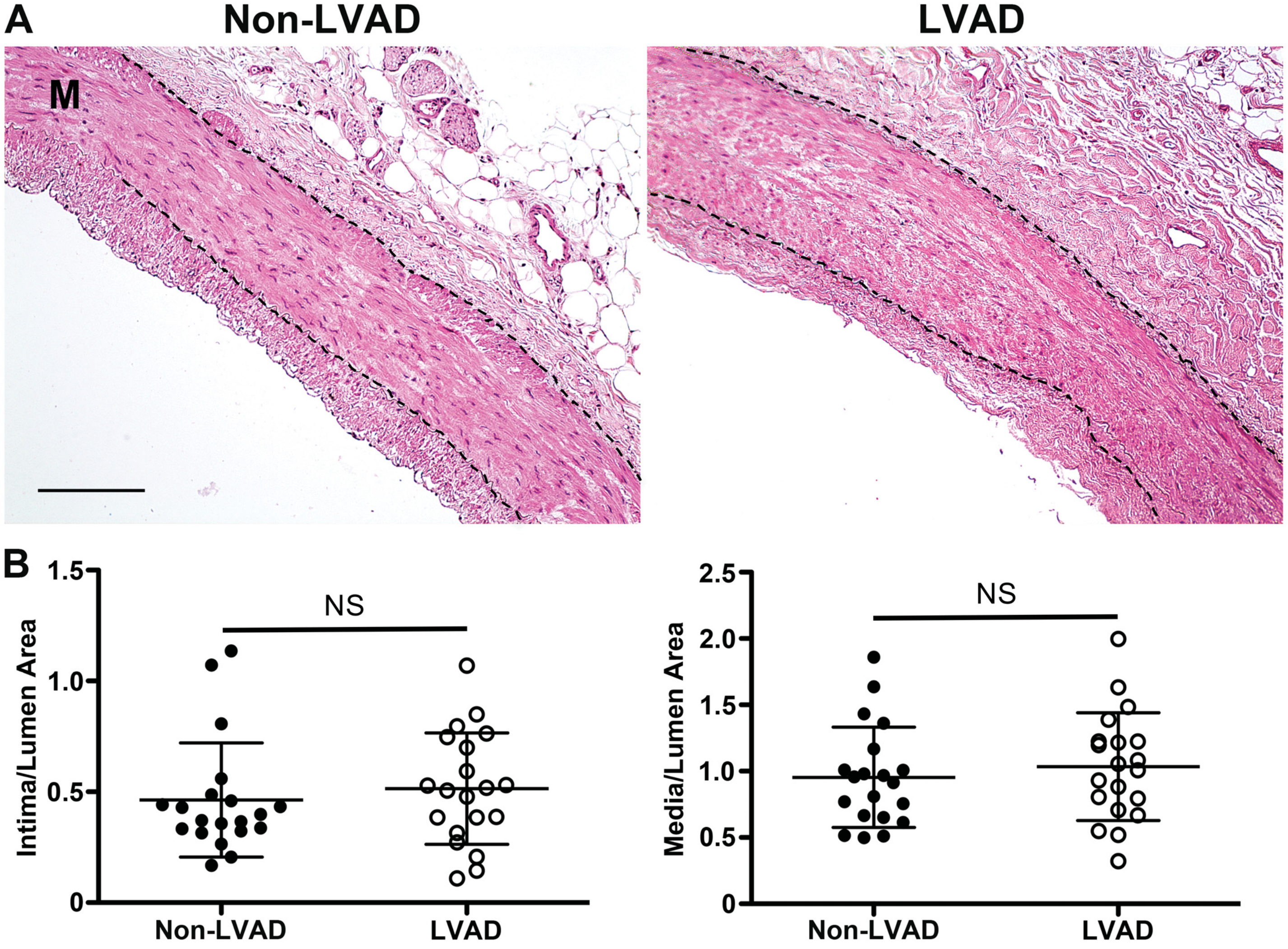

Supplemental Figure 3. Matched Intima and Media Thickness Between Coronary Arteries Exposed or not Exposed to Continuous Flow Left Ventricular Assist Devices (LVAD). Coronary arteries from explanted hearts of non-LVAD or CF-LVAD patients were matched for NAH. **A).** Representative H&E images showing similar intimal non-atherosclerotic hyperplasia in non-LVAD-exposed (left) compared CF-LVAD-exposed (right) vessels, and similar medial thickness when normalized to lumen diameter. Dashed lines delineate the arterial media; M = arterial media; I = arterial intima; scale bars = 100 μm. **B).** Intima areas (left graph) and media areas (right graph) of the coronary arteries were measured by Image J and normalized to the area of the vessel lumen to control for differences in vessel caliber. N=20 individual vessels per group. Compared to non-LVAD vessels, CF-LVAD vessels exhibited no change in relative intima or media areas (Two tailed Student's t tests, P=0.5305 for intima; P=0.5222 for media). Error bar represents mean ± standard deviation.

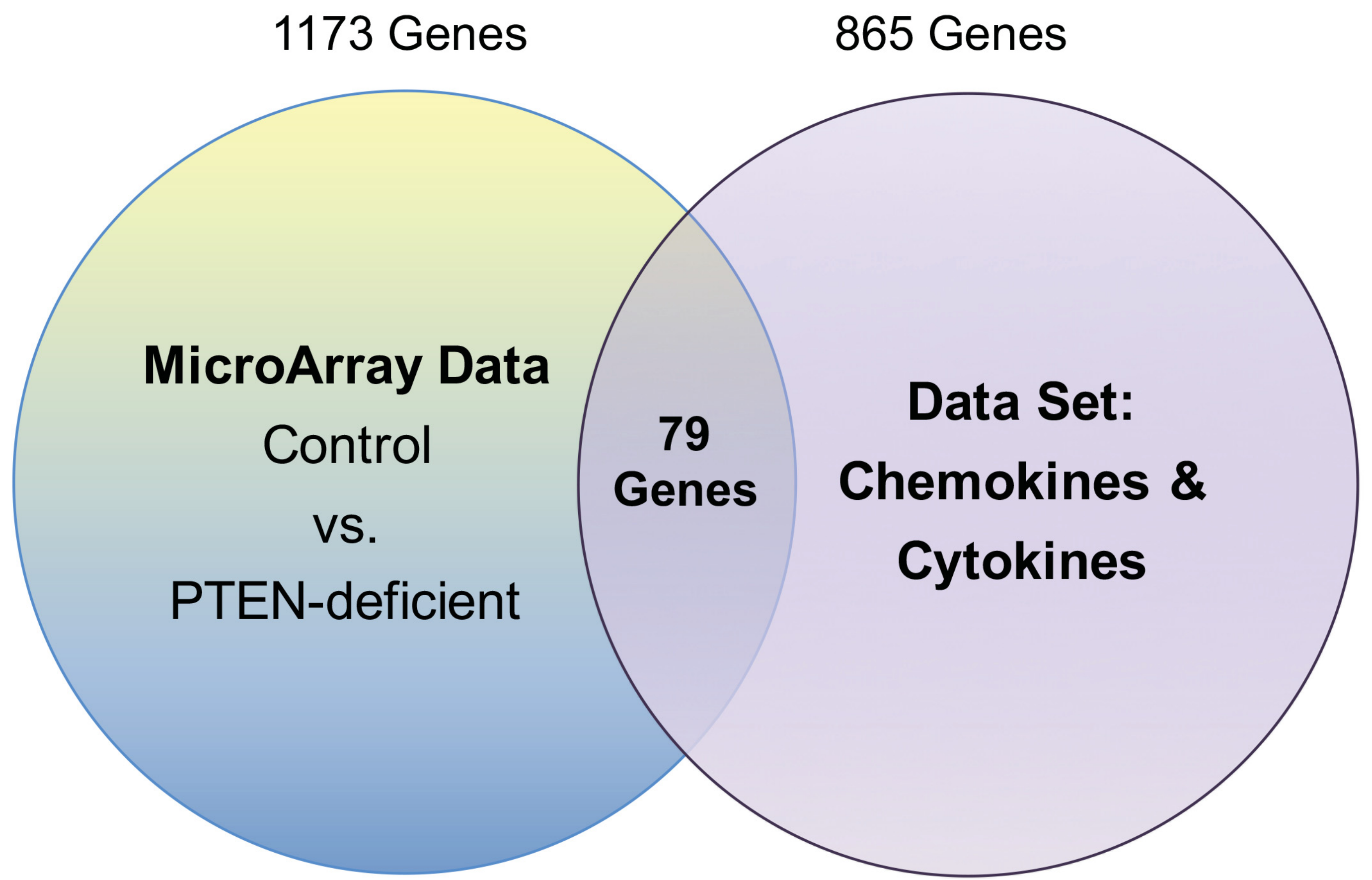

Supplemental Figure 4. Significantly Differentiated Genes Within Previously Published Datasets of Chemokine- and Cytokine-Associated Genes. 1173 differentially expressed genes were compared to previously published datasets of chemokine- and cytokine-associated genes. 79 genes were identified as commonly expressed in the PTEN-deficient SMC microarray and previously published chemokine / cytokine datasets.

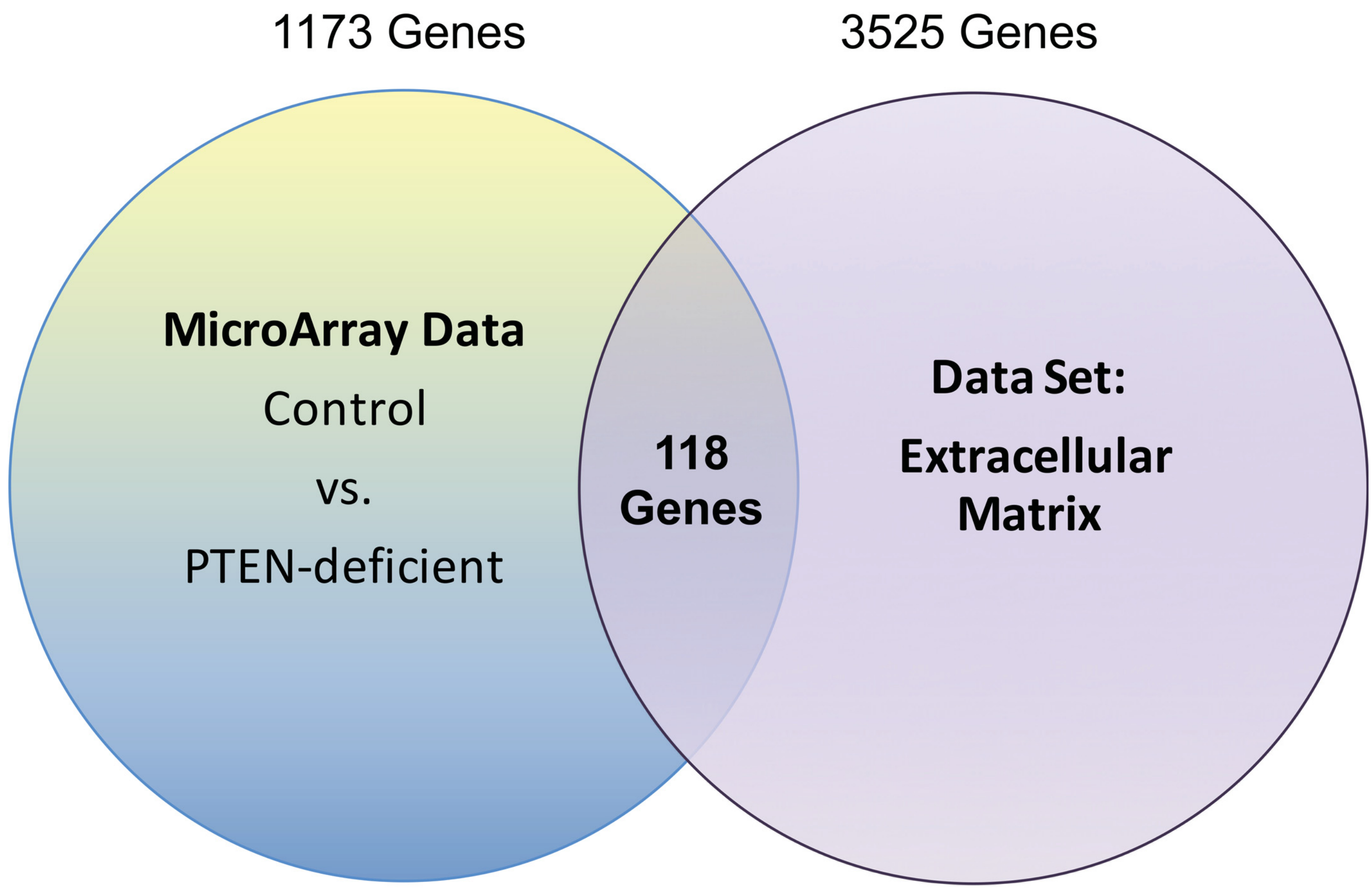

Supplemental Figure 5. Significantly Differentiated Genes Within Previously Published Datasets of Matrix-Associated Genes. 1173 differentially expressed genes were compared to previously published datasets of extracellular matrix- and matrix remodeling-associated genes. 118 genes were identified as commonly expressed in the PTEN-deficient SMC microarray and previously published extracellular matrix datasets.

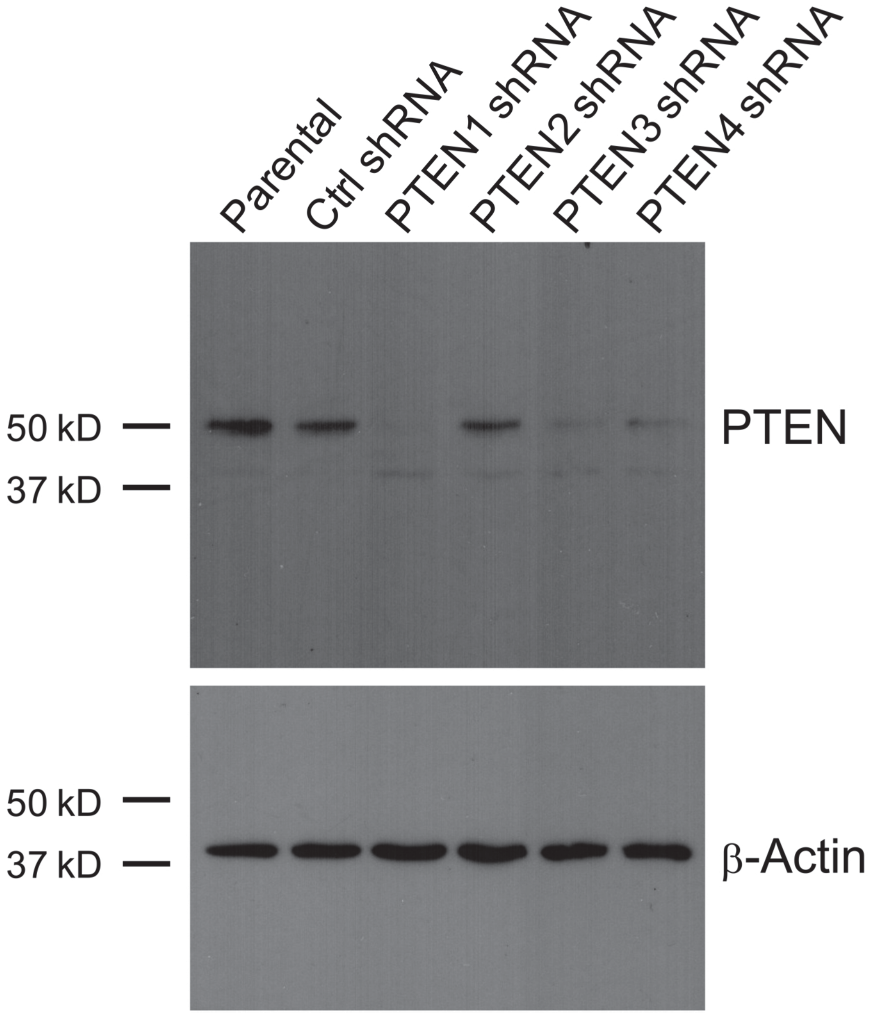

Supplemental Figure 6. Representative Western blot showing decreased PTEN expression in PTEN-deficient Smooth Muscle Cells (SMCs). Pools of SMCs stably expressing control (Ctrl) or PTEN-specific shRNA were serum-restricted for 72 h. Whole cell lysates were analyzed for total PTEN levels. Parental (non-infected) SMCs were used as a control for shRNA viral infection. β-Actin was used as a loading control.

Supplemental Table 7: Cytokine-associated Upregulated Genes In PTEN-Deficient SMCs

Gene Symbol	Gene Accession	Gene Description
Acvr2a	NM_031571	activin A receptor, type IIA
Adcy8	NM_017142	adenylate cyclase 8 (brain)
Bmp2	NM_017178	bone morphogenetic protein 2
C1s	NM_138900	complement component 1, s subcomponent
C2	NM_172222	complement component 2
C3	NM_016994	complement component 3
C6	NM_176074	complement component 6
Ccl11	NM_019205	chemokine (C-C motif) ligand 11
Ccl2	NM_031530	chemokine (C-C motif) ligand 2
Ccl20	NM_019233	chemokine (C-C motif) ligand 20
Ccl7	NM_001007612	chemokine (C-C motif) ligand 7
Cd55	NM_022269	Cd55 molecule
Cfh	BC089845	complement factor H
Clcf1	NM_207615	cardiotrophin-like cytokine factor 1
Ctsk	NM_031560	cathepsin K
Cxcl1	NM_030845	chemokine (C-X-C motif) ligand 1
Cxcl13	NM_001017496	chemokine (C-X-C motif) ligand 13
Cxcl3	NM_138522	chemokine (C-X-C motif) ligand 3
Cxcl6	NM_022214	chemokine (C-X-C motif) ligand 6
Dnm1	NM_080689	dynamin 1
Dusp6	NM_053883	dual specificity phosphatase 6
Fgf1	NM_012846	fibroblast growth factor 1
Fgf7	NM_022182	fibroblast growth factor 7
Fgfr2	NM_012712	fibroblast growth factor receptor 2
Gnai1	NM_013145	guanine nucleotide binding protein, alpha inhibiting 1
Gng8	NM_139185	guanine nucleotide binding protein, gamma 8
Hgf	NM_017017	hepatocyte growth factor
Il13ra1	NM_145789	interleukin 13 receptor, alpha 1
Il18bp	NM_053374	interleukin 18 binding protein
Il1f10	NM_001108571	interleukin 1 family, member 10
Il1f9	NM_001113790	interleukin 1 family, member 9
Il1r2	NM_053953	interleukin 1 receptor, type II
Il1rap	NM_012968	interleukin 1 receptor accessory protein
Il1rn	NM_022194	interleukin 1 receptor antagonist
Il20rb	DQ222846	interleukin 20 receptor beta
Il6	NM_012589	interleukin 6
Inhba	NM_017128	inhibin beta-A
Jak2	NM_031514	Janus kinase 2
Kitlg	NM_021843	KIT ligand
Lbp	NM_017208	lipopolysaccharide binding protein
Lgals3	NM_031832	lectin, galactoside-binding, soluble, 3
Nfkb1	ENSRNOT00000036838	nuclear factor kappa light polypeptide gene enhancer in B-cells 1
Nod1	NM_001109236	nucleotide-binding oligomerization domain containing 1
Osmr	NM_001005384	oncostatin M receptor
Pdgfc	NM_031317	platelet derived growth factor C
Pros1	NM_031086	protein S (alpha)
Sdf2l1	NM_001109433	stromal cell-derived factor 2-like 1
Shc4	ENSRNOT00000011084	SHC (Src homology 2 domain containing) family, member 4
Tlr2	NM_198769	toll-like receptor 2
Tnfrsf12a	NM_181086	tumor necrosis factor receptor superfamily, member 12a
Tnfrsf1b	NM_130426	tumor necrosis factor receptor superfamily, member 1b
Tnfrsf22	XM_001066154	tumor necrosis factor receptor superfamily, member 22
Tnfrsf9	NM_001025773	tumor necrosis factor receptor superfamily, member 9
Ube2l6	NM_001024755	ubiquitin-conjugating enzyme E2L 6
Vegfa	NM_031836	vascular endothelial growth factor A

Supplemental Table 8: Cytokine-associated Downregulated Genes In PTEN-Deficient SMCs

Gene Symbol	Gene Accession	Gene Description
Adcy2	NM_031007	adenylate cyclase 2 (brain)
Adcy3	NM_130779	adenylate cyclase 3
Akt3	NM_031575	v-akt murine thymoma viral oncogene homolog 3
Bcl2	NM_016993	B-cell CLL/lymphoma 2
Cx3cl1	NM_134455	chemokine (C-X3-C motif) ligand 1
Dkk3	NM_138519	dickkopf homolog 3 (Xenopus laevis)
Fos	NM_022197	FBJ osteosarcoma oncogene
Ikbkb	NM_053355	inhibitor of kappa light polypeptide gene enhancer in B-cells
Ikbkg	NM_199103	inhibitor of kappaB kinase gamma
Il15	NM_013129	interleukin 15
Il1rl1	NM_001127689	interleukin 1 receptor-like 1
Il33	NM_001014166	interleukin 33
Lifr	NM_031048	leukemia inhibitory factor receptor alpha
Map3k1	NM_053887	mitogen activated protein kinase kinase kinase 1
Mapkapk3	NM_001012127	mitogen-activated protein kinase-activated protein kinase 3
Met	NM_031517	met proto-oncogene
Plcb4	NM_024353	phospholipase C, beta 4
Rock1	NM_031098	Rho-associated coiled-coil containing protein kinase 1
Tgfb2	NM_031131	transforming growth factor, beta 2
Tgfb3	NM_013174	transforming growth factor, beta 3
Tnfrsf11b	NM_012870	tumor necrosis factor receptor superfamily, member 11b
Tnfsf18	ENSRNOT00000039221	tumor necrosis factor (ligand) superfamily, member 18
Txnip	NM_001008767	thioredoxin interacting protein
Vegfc	NM_053653	vascular endothelial growth factor C

Supplemental Table 9: ECM-associated Upregulated Genes In PTEN-Deficient SMCs

Gene Symbol	Gene Accession	Gene Description
Adam12	BC167765	ADAM metallopeptidase domain 12
Adamts4	AB042272	ADAM metallopeptidase with thrombospondin type 1 motif, 4
Adamts8	NM 001106811	ADAM metallopeptidase with thrombospondin type 1 motif, 8
Adamtsl1	XM 001061448	ADAMTS-like 1
Adamtsl3	NM 001107533	ADAMTS-like 3
Aebp1	NM 001100970	AE binding protein 1
Angpt1	NM 053546	angiopoietin 1
B3gat3	NM 001128184	beta-1,3-glucuronyltransferase 3 (glucuronosyltransferase I)
Cd109	NM 001108771	CD109 molecule
Clec11a	NM 001012459	C-type lectin domain family 11, member a
Clu	NM 053021	clusterin
Col15a1	ENSRNOT00000017217	collagen, type XV, alpha 1
Col4a1	NM 001135009	collagen, type IV, alpha 1
Col4a2	XM 001076134	collagen, type IV, alpha 2
Col6a3	NM_001109008	procollagen, type VI, alpha 3
Colec12	NM 001025721	collectin sub-family member 12
Ctsh	NM 012939	cathepsin H
Dcn	NM_024129	decorin
Egfl7	NM 139104	EGF-like-domain, multiple 7
Esm1	NM 022604	endothelial cell-specific molecule 1
Fam20c	NM 001012238	family with sequence similarity 20, member C
Fbln1	NM 001127547	fibulin 1
Fndc1	NM 001038615	fibronectin type III domain containing 1
Frzb	NM 001100527	frizzled-related protein
Fst	NM 012561	follistatin
Grem1	NM 019282	gremlin 1, cysteine knot superfamily, homolog (Xenopus laevis)
Has1	NM 172323	hyaluronan synthase 1
Hpse	NM_022605	heparanase
Hpx	NM 053318	hemopexin
Htra1	NM 031721	HtrA serine peptidase 1
Htra3	ENSRNOT00000010852	HtrA serine peptidase 3
Itga7	NM 030842	integrin alpha 7
Itgb8	NM 001108726	integrin beta 8
Lama4	NM_001107635	laminin, alpha 4
Loxl4	NM 001107592	lysyl oxidase-like 4
Ltbp1	NM 021587	latent transforming growth factor beta binding protein 1
Lum	NM 031050	lumican
Mgp	NM 012862	matrix Gla protein
Mmp19	NM 001107159	matrix metallopeptidase 19
Mmp2	NM 031054	matrix metallopeptidase 2
Nid2	NM 001012005	nidogen 2
Ntn1	NM 053731	netrin 1
Plod1	NM 053827	procollagen-lysine 1, 2-oxoglutarate 5-dioxygenase 1
Podnl1	ENSRNOT00000037453	podocan-like 1
Prg4	NM 001105962	proteoglycan 4, (megakaryocyte stimulating factor)syndrome)
Sema3a	NM 017310	short basic domain, secreted, (semaphorin) 3A
Sema3c	NM 001106578	short basic domain, secreted, (semaphorin) 3C
Sema3e	NM 001106579	short basic domain, secreted, (semaphorin) 3E
Serpinb7	NM 130404	serine (or cysteine) peptidase inhibitor, clade B, member 7
Serpine2	NM 019197	serine (or cysteine) peptidase inhibitor, clade E, member 2
Serping1	NM 199093	serine (or cysteine) peptidase inhibitor, clade G, member 1
Serpini1	NM 053779	serine (or cysteine) peptidase inhibitor, clade I, member 1
Sfrp2	NM 001100700	secreted frizzled-related protein 2
Sfrp4	NM 053544	secreted frizzled-related protein 4
Slpi	NM 053372	secretory leukocyte peptidase inhibitor
Srpx	NM 022524	sushi-repeat-containing protein, X-linked
Sv2a	NM 057210	synaptic vesicle glycoprotein 2a
Tgfa	NM 012671	transforming growth factor alpha
Timp1	NM 053819	TIMP metallopeptidase inhibitor 1
Tnn	NM 001107189	tenascin N
Vcan	AF072892	versican
Vwa5a	NM 198755	von Willebrand factor A domain containing 5A

Supplemental Table 10: ECM-associated Downregulated Genes In PTEN-Deficient SMCs

Gene Symbol	Gene Accession	Gene Description
Adamts12	NM_001106420	ADAM metallopeptidase with thrombospondin type 1 motif, 12
Adamts9	NM_001107877	ADAM metallopeptidase with thrombospondin type 1 motif, 9
Angptl2	NM_133569	angiopoietin-like 2
Anxa8	NM_001031654	annexin A8
Aspn	NM_001014008	asporin
Bmp3	NM_017105	bone morphogenetic protein 3
Bmp4	NM_012827	bone morphogenetic protein 4
Bmp6	NM_013107	bone morphogenetic protein 6
Btc	NM_022256	betacellulin
Cav1	NM_031556	caveolin 1, caveolae protein
Cav2	NM_131914	caveolin 2
Col11a1	ENSRNOT00000024138	collagen, type XI, alpha 1
Col4a5	ENSRNOT00000025677	collagen, type IV, alpha 5
Crispld2	NM_138518	cysteine-rich secretory protein LCCL domain containing 2
Ctgf	NM_022266	connective tissue growth factor
E2f5	ENSRNOT00000014361	E2F transcription factor 5
Eln	NM_012722	elastin
Ereg	NM_021689	epiregulin
Fbln2	ENSRNOT00000009696	fibulin 2
Fbln5	NM_019153	fibulin 5
Fmod	NM_080698	fibromodulin
Fras1	ENSRNOT00000002814	Fraser extracellular matrix complex subunit 1
Gdf6	NM_001013038	growth differentiation factor 6
Gpc4	NM_001014108	glypican 4
Hmcn1	ENSRNOT00000030951	hemicentin 1
Hmmr	NM_012964	hyaluronan mediated motility receptor (RHAMM)
Hs6st1	NM_001108210	heparan sulfate 6-O-sulfotransferase 1
Id2	NM_013060	inhibitor of DNA binding 2
Itga1	NM_030994	integrin alpha 1
Itga11	NM_001108156	integrin, alpha 11
Itga4	NM_001107737	integrin alpha 4
Itga6	ENSRNOT00000045394	integrin, alpha 6
Matn2	ENSRNOT00000008361	matrilin 2
Megf9	NM_001107940	multiple EGF-like-domains 9
Mfap4	NM_001034124	microfibrillar-associated protein 4
Mfap5	NM_001108644	microfibrillar associated protein 5
Mmp28	NM_001079888	matrix metallopeptidase 28 (epilysin)
Nrg1	NM_031588	neuregulin 1
Omd	NM_031817	osteomodulin
P4ha3	NM_198775	procollagen-proline 4-hydroxylase, alpha polypeptide III
Pcsk5	ENSRNOT00000054793	proprotein convertase subtilisin/kexin type 5
Pdgfd	NM_023962	platelet-derived growth factor, D polypeptide
Pik3ip1	NM_001017453	phosphoinositide-3-kinase interacting protein 1
Plxna2	NM_001105988	plexin A2
Pxdn	ENSRNOT00000060139	peroxidasin homolog (Drosophila)
S100a10	NM_031114	S100 calcium binding protein A10
Sfrp1	ENSRNOT00000024128	secreted frizzled-related protein 1
Slit3	NM_031321	slit homolog 3 (Drosophila)
Smad6	NM_001109002	SMAD family member 6
Smad7	NM_030858	SMAD family member 7
Smoc1	NM_001002835	SPARC related modular calcium binding 1
Smurf2	NM_001107061	SMAD specific E3 ubiquitin protein ligase 2
Spon1	NM_172067	spondin 1, extracellular matrix protein
Tinagl1	NM_053582	tubulointerstitial nephritis antigen-like 1
Tll1	NM_001106081	tolloid-like 1
Wisp2	NM_031590	WNT1 inducible signaling pathway protein 2

Supplemental Table 11: Cytokine and ECM-associated Genes Upregulated 1.8-2.0-fold in PTEN-Deficient SMCs

Gene Symbol	Gene Accession	Gene Description
Adamts6	NM_001108544	ADAM metallopeptidase with thrombospondin type 1 motif, 6
Angptl4	NM_199115	angiopoietin-like 4
Ccr1	NM_020542	chemokine (C-C motif) receptor 1
Col6a2	ENSRNOT00000001695	collagen, type VI, alpha 2
Ctsb	NM_022597	cathepsin B
Cxcl12	NM_001033883	chemokine (C-X-C motif) ligand 12
Efemp2	NM_001005907	EGF-containing fibulin-like extracellular matrix protein 2
Foxo3	NM_001106395	forkhead box O3
Gdf15	NM_019216	growth differentiation factor 15
Gnb4	NM_001013910	guanine nucleotide binding protein, beta polypeptide 4
Hif1a	NM_024359	hypoxia-inducible factor 1, alpha subunit
Il17b	ENSRNOT00000026679	interleukin 17B
Il34	NM_001025766	interleukin 34
Il4ra	NM_133380	interleukin 4 receptor, alpha
Itga3	NM_001108292	integrin alpha 3
Itgb5	NM_147139	integrin, beta 5
Kng1	NM_012696	kininogen 1
Lama2	ENSRNOT00000014917	laminin, alpha 2
Lamb1	NM_001106721	laminin, beta 1
Lamb3	ENSRNOT00000008440	laminin, beta 3
Loxl3	NM_001107866	lysyl oxidase-like 3
Pik3r1	NM_013005	phosphoinositide-3-kinase, regulatory subunit 1 (alpha)
Slit2	AF141386	slit homolog 2 (Drosophila)
Tgfb1	NM_021578	transforming growth factor, beta 1
Tgm2	NM_019386	transglutaminase 2, C polypeptide
Thbs2	ENSRNOT00000014552	thrombospondin 2
Tnfrsf10b	NM_001108873	tumor necrosis factor receptor superfamily, member 10b
